# Importance of Monomer-Flexibility Effects for Spectra of Molecular Clusters

**DOI:** 10.1021/acs.jpclett.6c00742

**Published:** 2026-04-20

**Authors:** Marcin Stachowiak, Ewelina Grabowska, Xiao-Gang Wang, Tucker Carrington, Krzysztof Szalewicz, Piotr Jankowski

**Affiliations:** † Faculty of Chemistry, 49577 Nicolaus Copernicus University in Toruń, Gagarina 7, 87-100 Toruń, Poland; ‡ Chemistry Department, 4257 Queen’s University, Kingston, Ontario K7L 3N6, Canada; ¶ Department of Physics and Astronomy, 5972 University of Delaware, Newark, Delaware 19716, United States

## Abstract

A global full-dimensional description of interactions in a molecular van der Waals cluster, including both inter and intramolecular degrees of freedom, may seem to be the necessary starting point for high-accuracy nuclear dynamics calculations. Such calculations are currently able to make predictions for clusters, molecular collisions, and condensed phases accurate enough to be confronted with experiment. However, the all-dimensional treatment becomes prohibitively expensive for clusters with more than 6 atoms due to the “curse of dimensionality”. On the other hand, the rigid-monomer approximation allows applications to much larger clusters. We show on the example of H_2_–CO that if the rigidity is imposed via averaging over monomer vibrations, the predictions from such a reduced-dimensionality model can be about as accurate as those from the full-dimensional one; in fact, here both models predict spectra equally well. Moreover, we show that an approximate version of such an averaged surface, based on the Taylor expansion, which does not require the development of a full-dimensional surface and is affordable for larger molecules, also works very well. In contrast, models based on frozen geometries of monomers work much worse. Spectral and scattering calculations with the vibrationally averaged reduced-dimensionality models will result in insights into soft condensed matter properties, cold and ultracold molecular collisions, and physics of cold interstellar clouds that are currently not possible.

## Graphical abstract

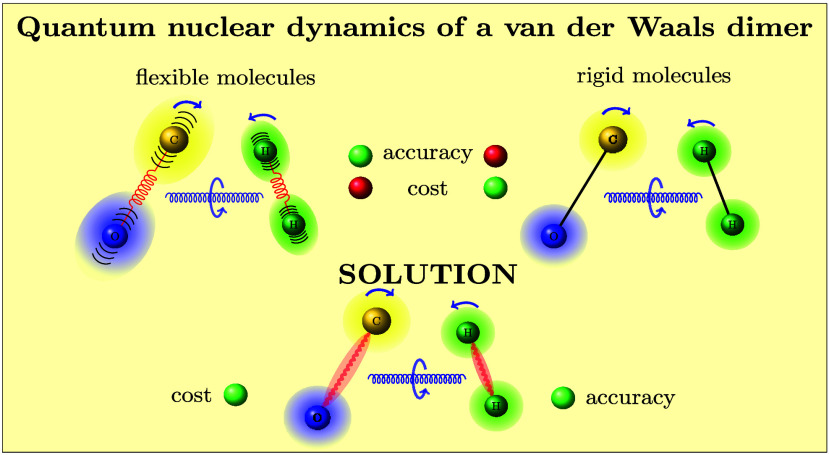

Intermolecular or van der Waals forces (interactions) determine the behavior of clusters of molecules, molecular condensed phases, and biomolecular systems. Not that long ago, the only quantitative description of such interactions was provided by inversions of experimental data, leading to so-called empirical force fields (empFFs). However, currently the FFs created from first-principles, i.e., by fitting a functional form to results of *ab initio* calculations (aiFFs), mostly replaced empFFs in investigations clusters with small monomers, in particular in nuclear dynamics calculations of rovibrational spectra and scattering cross sections. Also in molecular dynamics (MD) simulations of matter, aiFFs are becoming increasingly popular, in particular with the rapid progress in developing machine-learned FFs fitted to *ab initio* data. The predictions of the best aiFFs are significantly more accurate than those of any empFFs. In many cases, the properties of matter derived from aiFFs not only agree very well with experimental results, sometimes getting close to experimental accuracy, but can be used to interpret and complement experiments. The conceptual importance of such results is that the ability to predict properties of arbitrary molecular matter from first principles, directly from equations of quantum mechanics, leads to the deepest possible physical insights and to novel predictions of importance for science and technology. One such example are crystal structure predictions for rigid monomers.^1^

The major barrier that does not allow full-dimensional applications of aiFFs to monomers much larger than triatomics is the tensor product “curse of dimensionality”, i.e., the *k* ^*D*^ scaling of the number of points needed to fit a surface or to perform nuclear dynamics calculations, where *k* is the number of points per dimension and *D* is the number of degrees of freedom. We show that this barrier can be overcome without a significant decrease in accuracy of predictions by using reduced-dimensionality approaches based on potential energy surfaces (PES) averaged over the vibrations of the monomers. While we will concentrate on applications to rovibrational spectra due to the existence of experimental results that allow rigorous testing of the proposed methodology, this methodology applies equally well to molecular collisions. Therefore, it will be of importance in studies of cold and ultracold collisions involving molecules, an emerging frontier in physics owing to the rich intramolecular rovibrational structure, holding promise of physical insights and applications that are not possible if only atoms are involved.^2–9^ Interactions between cold molecules are also relevant for astrophysics.^10–20^ While for atom–diatom interactions a full-dimensional treatment is currently straightforward, for diatom–diatom interactions or interactions involving larger molecules^6,7,18,19,21–45^ dimensionality becomes an issue. However, if the rigid-monomer approximation is used, the dimensionality is greatly reduced, for example, for the water trimer from 21 to 12 degrees of freedom (DoFs). In fact, for dimers the dimensionality is 6 at the most. With such dramatically reduced dimensionality, the number of grid points, i.e., configurations of clusters for which *ab initio* calculations have to be performed, is also dramatically reduced. On the other hand, one may expect that the accuracy of predictions will also be significantly reduced. The present work examines various methods of imposing rigid-monomer approximation to determine ways of minimizing the reduction of accuracy and proposes a relatively inexpensive yet accurate reduction method.

There is surprisingly little information in literature on how much theoretical predictions are improved upon moving from a rigid-monomer approximation to full-dimensional treatment. There are numerous papers employing each approach, but only a handful of papers that compared various dimensionality treatments on equal footing.^18,34,45–47^ The reduced-dimensionality projects usually did not have appropriate software and computer power to perform full-dimensional calculations. The reason that the full-dimensional projects did not include reduced-dimensionality calculations (which can be obtained with little extra costs) was probably the perception that results of such calculations would have significantly reduced accuracy.

The simplest way to reduce dimensionality is to set internal coordinates to some values, in this way creating “frozen” monomers. Most of reduced-dimensionality calculations used monomers fixed at their equilibrium geometry. One can also reduce the dimensionality by averaging full-dimensional PESs over the vibrational wave functions of monomers. Note that the latter reduced-dimensionality PESs do not correspond to any fixed monomer geometry, so they cannot be called frozen monomers PESs. The concept of averaging was apparently used for the first time by Le Roy and van Kranendonk^48^ in 1974 for Rg–H_2_/D_2_ dimers, even before the development of first aiFFs (the PES obtained by these authors was empirical, i.e., fitted to spectra of these dimers). The first aiFFs with dimensionality reduced by averaging were developed in the early to mid 2000s: for Ar–HF (3D to 2D) in ref 46, for H_2_–CO (6D to 4D) in ref 49, and for H_2_–H_2_O (9D-5D) in ref 50. A more recent example are calculations for N_2_–HF (6D to 4D) in ref 51. These reduced dimensionality PESs were used in rovibrational calculations, and when confronted with experimental data, usually lead to very good agreement, but based on these results one cannot judge the accuracy of the averaging procedure since the agreement could potentially be much better with the full-dimensional PESs. Our investigations presented here are aimed at answering these questions.

The performance of reduced-dimensionality approximations obviously depends on the strength of intermolecular (noncovalent) interactions, which varies from a few (He–He) to a few thousands (formic acid dimer) cm^–1^. Here, we will investigate the dimensionality issues on the example of H_2_–CO, a complex with a relatively small magnitude of interaction energy, of the order of 100 cm^–1^. For this complex, of great importance for astrophysics,^11–18,20^ a very accurate six-dimensional (6D) PES has recently been published^52^ and it can be used to study various reduced-dimensionality approximations. More details about that surface, dubbed *V*_23_ in ref 52 and called *V* in the present paper, are given in Potential Energy Surfaces in Methods. To parametrize the geometry of H_2_–CO, we use the Jacobi coordinates defined in the Supporting Information (SI), Sec. SII. Thus, the surface depends on four intermolecular coordinates: the distance *R* between the centers of mass (COMs) of monomers and the three angles θ_1_, θ_2_, and ϕ, as well as two intramolecular coordinates: interatomic distances *r*_1_ and *r*_2_ in H_2_ and CO, respectively. Investigations of H_2_–CO in ref 52 concentrated on 6D treatment, and although 4D results were used for a part of the spectrum, no comprehensive comparisons of the two approaches were attempted. Such comparisons are the subject of the present work. We will test three different methods employing averaging-based dimensionality reductions in addition to two methods relying on freezing geometries of monomers.

## 4D Potential Energy Surfaces

We have investigated five 4D PES, i.e., depending only on the intermolecular coordinates, each one obtained using different approximations: (*a*) *V*(*r*_1*e*_, *r*_2*e*_), calculated with the monomers at their equilibrium geometries (the most popular way to construct the reduced-dimensionality surfaces due to the easy generation of such geometries via energy minimization in electronic structure packages); (*b*) *V*(⟨*r*_1_⟩_*v*_1__, ⟨*r*_2_⟩_*v*_2__), where the arguments ⟨*r*_1_⟩_*v*_1__ and ⟨*r*_2_⟩_*v*_2__ denote the values of *r*_1_ and *r*_2_ averaged over the *v*_1_ and *v*_2_ vibrational wave functions of H_2_ and CO, respectively (the approach introduced in ref 53 and later explored in ref 46, now becoming popular in applications to PESs for small monomers); (*c*) ⟨*V*⟩_*v*_1_ *v*_2__^TE^, a surface obtained by first replacing the PES by its Taylor expansion (TE) in *r*_1_ and *r*_2_ up to second-order terms and then averaging over vibrations of the isolated monomers, i.e., unperturbed by the complex formation^54–56^ (see Potential Energy Surfaces in Methods); (*d*) ⟨*V*⟩_*v*_1_ *v*_2__^TE3^, analogous to ⟨*V*⟩_*v*_1_ *v*_2__^TE^, but using third-order Taylor expansion (see Sec. SIV in SI); (*e*) ⟨*V*⟩_*v*_1_ *v*_2__, the directly averaged *V* over the vibrations of the isolated molecules (Potential Energy Surfaces in Methods). In the nuclear dynamics calculations performed with the BOUND program,^57,58^ all 4D surfaces were generated on-the-fly from the 6D surface *V*, i.e., the reduced dimensionality interaction energies were computed purely numerically, which allowed us to avoid potential inaccuracies introduced by the 4D fitting. The results presented here were obtained for both molecules in their ground vibrational states. However, we have also performed calculations for the complex with CO in the first excited state and the results show the same trends as discussed below. Notice that while here ⟨*V*⟩_*v*_1_ *v*_2__, ⟨*V*⟩_*v*_1_ *v*_2__^TE^, and ⟨*V*⟩_*v*_1_ *v*_2__^TE3^ were all obtained from 6D *V*, in future applications the latter two surfaces can be constructed without knowledge of the full-dimensional surface. This feature can extend the applicability of the averaged surfaces. The energies and geometries of the global and local minima and saddle points are given for all surfaces in Table SI in SI.

## Average Deviations of 4D vs 6D from Experiment

To evaluate the performance of the approximations investigated here, we computed the root-mean square errors (RMSEs) of rovibrational energy levels from 4D and 6D nuclear motion calculations relative to experiment. These results are shown in Figure 1 (see also Table SII **in SI**).

**Figure 1:**
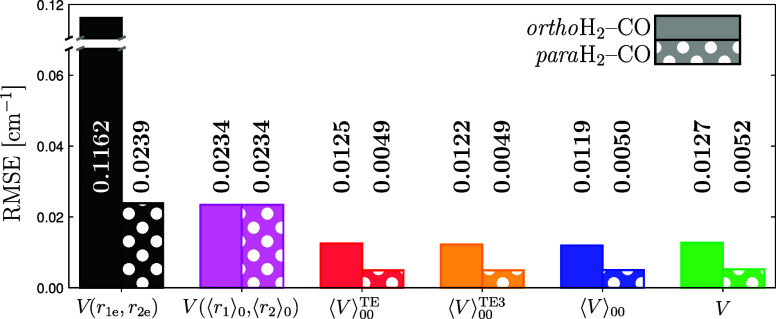
RMSEs of rovibrational energy levels (in cm^–1^) obtained from the 6D and 4D calculations with surfaces listed on the horizontal axis. The RMSEs are relative to the experimental levels given in ref 52. Only the bound states are included in this comparison. All rovibrational energies are measured from the lowest energy level of each method.

The first striking observation from these figures and tables is that the *V*(*r*_1*e*_, *r*_2*e*_) surface results have errors an order of magnitude larger than those of the 6D results. Clearly, this often used method of dimensionality reduction is the worst possible choice in particular since the 4D calculations using *V*(⟨*r*_1_⟩_0_, ⟨*r*_2_⟩_0_), which are exactly of the same cost, reduce the error of *V*(*r*_1*e*_, *r*_2*e*_) predictions by a factor of 5. The other striking observation are the very small differences in RMSEs between the 4D results based on averaging of *V* and the 6D results. These RMSEs range between 0.0119 and 0.0127 cm^–1^, very small errors relative to the values of the energy levels which are of the order of 10 cm^–1^. The RMSEs of levels computed with the ⟨*V*⟩_00_^TE^, ⟨*V*⟩_00_^TE3^, and ⟨*V*⟩_00_ surfaces decrease from 0.0125 through 0.0122 to 0.0119 cm^–1^, as expected, since the accuracy of the rigid-monomer approximation should increase in this sequence. However, the increases are very small, showing that already the simplest way of performing the averaging, leading to ⟨*V*⟩_00_^TE^, is sufficient. The surprising fact that the complete 6D treatment results in a slightly larger RMSE than the best 4D treatments is discussed in **Sec. SVI in** SI.

## Performance of 4D vs 6D for Individual Levels of *para*-H_2_–CO

The results for individual energy levels of *para*-H_2_–CO are plotted in Figure 2A (see also Table SIII **in SI**) as the differences of the rovibrational energies computed using a given method with respect to the experimental ones, Δ_method_ = *E*_method_^0^ – *E*_expt_^0^. A comparison of these results with those of Figure 1 sheds light on various aspects of both theory and experiment. First, notice that the deviations of 6D and 4D averaged-*V* theory from experiment, shown in the lower panel of Figure 2A, do not have any significant outliers. This shows that the experimental levels were correctly deduced from transition energies.^52,55,56,59^ Such confirmation is important since this deduction is highly nontrivial and for *ortho-*H_2_–CO it could be achieved only using theoretical guidance.^52,55,56^ Also, the very good agreement between ⟨*V*⟩_00_^TE^, ⟨*V*⟩_00_^TE3^, and ⟨*V*⟩_00_ and their correct convergence trends indicate the correctness of our implementations, whose technical realizations are quite different. On the other hand, these findings are also a confirmation of the correctness of large-scale, difficult-to-converge 6D calculations. Analogous observations and conclusions can be drawn from Figure 2B for the *ortho*-H_2_–CO case. There are further insights from Figure 2A. In the upper panel, one can see very large Δ_*r*_e__ errors. In particular, for two states the errors are extremely large and they actually determine the overall RMSE. The Δ_⟨*r*⟩_ errors are of a more uniform magnitude, but are overall significantly larger than the Δ_6D_ errors. The experimental uncertainty is much smaller than the latter deviations, less than 0.001 cm^–1^ (see ref 52). In some cases, the Δ_*r*_e__ or Δ_⟨*r*⟩_ errors are very small, sometimes essentially zero, and much smaller than the Δ_6D_ ones, which is clearly accidental. The Δ_⟨*r*⟩_ errors are mostly positive. In contrast, the Δ_*r*_e__ errors behave somewhat erratically (despite similar RMSEs for *para*-H_2_–CO in both cases). Due to this behavior, transition energies would be much more accurate in the former case. In the lower panel of Figure 2A, one can find a comparisons including Δ_TE_, Δ_TE3_, Δ_av_, and Δ_6D_. Notice a different vertical scale of this panel than on the upper one. A striking observation is that all these approaches give essentially identical errors.

**Figure 2:**
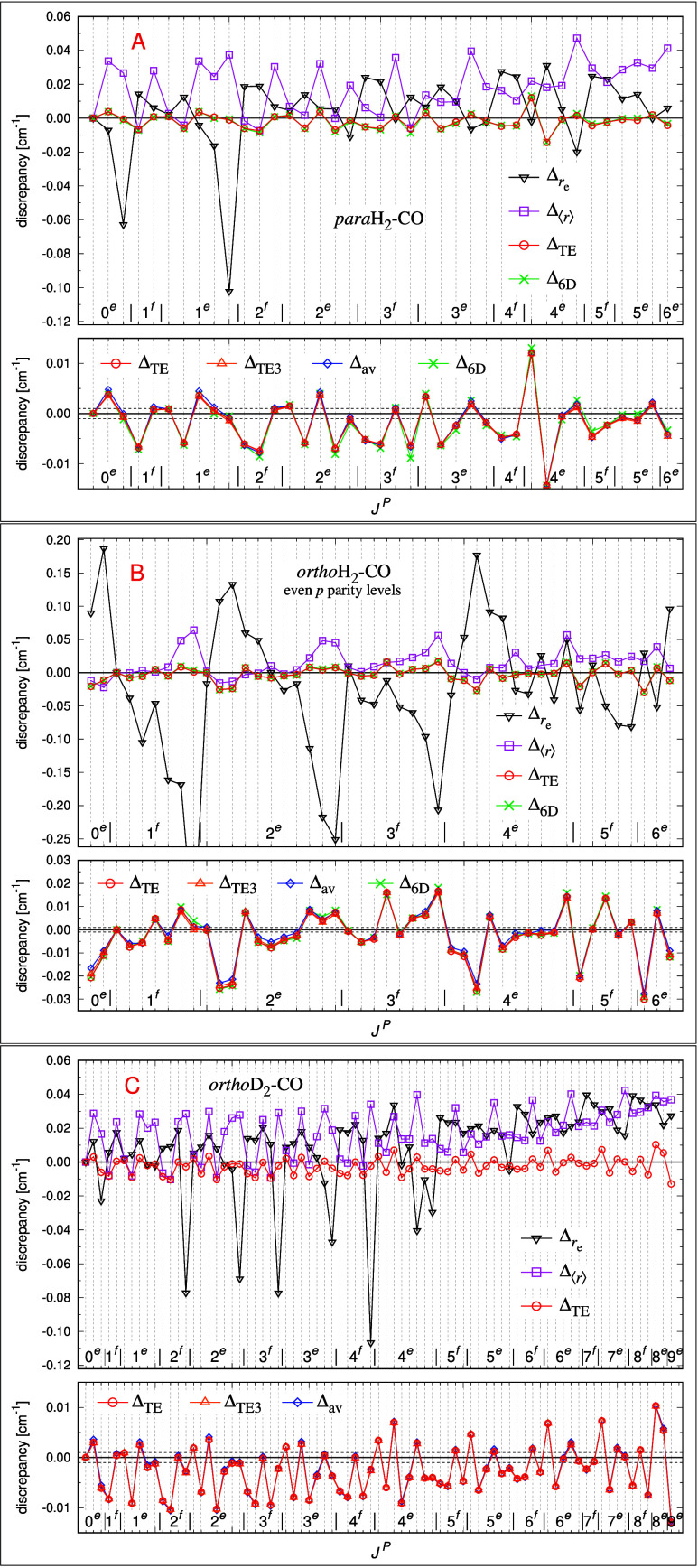
Comparison of the bound rovibrational energy levels of *para*-H_2_–CO (part A), even *p* parity levels of *ortho*-H_2_–CO (part B), and *ortho*-D_2_–CO (part C), calculated in various 4D approximations. The figures show the errors of the levels computed using a given method with respect to the experimental ones: Δ_method_ = *E*_method_^0^ – *E*_expt_^0^. Each entry on the horizontal axis represents an energy level and they are ordered according to their ascending value within each *J* ^*P*^ symmetry block, where *J* is the rotational quantum number and *P* is the spectroscopic parity. The vertical lines facilitate tracking the individual points. The horizontal dashed lines in lower panels mark the uncertainty of the experimental levels.

## Performance of 4D vs 6D for Individual Levels of *ortho*-H_2_–CO

A similar comparison for the *ortho*-H_2_–CO even *p* parity levels is presented in Figure 2B (see also Table SV in SI). Analogous data for the odd *p* levels are in Figure S2 and Table SVI in SI. As it can be seen in Figure 2B, the accuracy of the 4D calculations with the *V*(*r*_1*e*_, *r*_2*e*_) surface is much worse than in the case of *para*-H_2_–CO (notice different scales of the upper panels of these figures), which correlates with the very large RMSE. Thus, this surface does not predict reasonable spectra for *ortho*-H_2_–CO. One can see that while the Δ_*r*_e__ errors may seem to be tiny in comparison to the infrared transition energies, they are larger than the separations between experimental lines. The accuracy of the levels computed from the *V*(⟨*r*_1_⟩_0_, ⟨*r*_2_⟩_0_) surface is the same (in terms of RMSE, see Figure 1) as for the *para*-H_2_–CO case. The values of Δ_⟨*r*⟩_ are again mostly positive. Although *V*(⟨*r*_1_⟩_0_, ⟨*r*_2_⟩_0_) gives much more accurate levels than *V*(*r*_1*e*_, *r*_2*e*_) for *ortho-*H_2_–CO, it is still not accurate enough for a complete assignment of spectra. Similarly as in the *para* case, the errors plotted in the lower panel of Figure 2B are almost identical at the scale of the figure and are much smaller than the Δ_⟨*r*⟩_ errors. The slightly better performance of ⟨*V*⟩_00_ than of *V* discussed above is actually visible in the lower panel of Figure 2B: the blue points are quite systematically closer to experiment than the green ones. This shows that the difference in RMSEs does not result from a couple of outliers.

The errors presented in Figures 1 and 2, were relative to experiment. One can also compare various approximations relative to the 6D results, the highest level of theory. Such comparisons are presented in **Tables SIV and SVII in** SI. The results in these tables confirm that the 4D energy levels calculated from the ⟨*V*⟩_00_^TE^, ⟨*V*⟩_00_^TE3^, and ⟨*V*⟩_00_ surfaces are all very close to the corresponding 6D energies. This means that for H_2_–CO the effect of the monomers’ vibrations on the interaction energy is well captured already in the TE approximation. The closeness of the ⟨*V*⟩_00_^TE^ 4D results to the 6D ones is especially encouraging since, as discussed earlier, ⟨*V*⟩_00_^TE^ can be constructed at a cost comparable to the rigid monomer surface if the derivatives in eq 1 (see Methods) are calculated with a less expensive method than the leading term.^54,55^

## Performance of 4D Approximations for *ortho*-D_2_–CO

Additional test of the methods discussed can be made on dimers with isotopic substitution. The rovibrational energies of *ortho*-D_2_–CO obtained for all 4D surfaces studied here are compared with the experimental energies^60^ in Figure 2C (see also Tables SII and SVIII in SI). It can be seen that the observations of performance of the 4D methods made for H_2_–CO are confirmed: the results obtained from ⟨*V*⟩_00_^TE^, ⟨*V*⟩_00_^TE3^ and ⟨*V*⟩_00_ are very close to each other and close to the experimental values; the corresponding RMSEs are 0.0053, 0.0054, and 0.0054 cm^–1^. Also consistently, the surfaces based on rigid monomers, *V*(*r*_1*e*_, *r*_2*e*_) and *V*(⟨*r*_1_⟩_0_, ⟨*r*_2_⟩_0_), give much less accurate energies, with RMSEs of 0.0281 and 0.0221 cm^–1^, respectively.

## Scattering States

Since only experimental spectral data provide sufficiently rigorous evaluations of the methods investigated here, we restricted comparisons so far to bound states, although as already mentioned these methods are equally applicable to molecular collisions. Although, measurements of collisional properties have generally much larger uncertainties than spectral measurements, as an exception, in the case of H_2_–CO very precise information about the low-lying scattering resonances is available from spectral measurements. Comparisons of such data with results from scattering calculations (performed with the MOLSCAT program^61,62^ ) using various 4D approximations, are displayed in Table SIX in SI. The RMSEs are consistent with those for the bound states and the hierarchy of predictions quality is conserved. Obviously, the reduced-dimensionality approaches are not suitable for reactive collisions, where the explicit treatment of the intramolecular degrees of freedom is necessary.

## Comparisons with Literature

As demonstrated above, the accuracy of the 4D spectra obtained with any averaged-*V* method and that of the corresponding 6D spectra are essentially the same for H_2_–CO. An important question is how this finding extrapolates to other dimers. As already mentioned, there are many small dimers for which either full- or reduced-dimensionality calculations of rovibrational levels have been published. There is also a number of papers were both approaches were used, for example, in the work of Bačić, Felker, and collaborators^32,63,64^ and Wang and Carrington^36,40^ reduced-dimensionality calculations are always used to develop basis sets for the full-dimensionality ones. However, in none of these papers is there sufficient information on the three types of results needed to make meaningful comparisons: (*a*) reduced-dimensionality; (*b*) full-dimensionality; (*c*) experimental. To our knowledge, there are only two papers that compared the two approaches on an equal footing.^34,46^ We were able to obtain one more such comparison by combining results from refs 36, 38, 65, and 66 , see Table SX in SI. Note that results of ref 47 cannot be used since the versions of the HHB PESs used in ref 47 and in 6D+6D calculations of ref 67 are different. Table SX shows that, for both Ar–HF and (H_2_O)_2_, the difference of RMSEs between the ⟨*V*⟩ and full dimensionality approaches is negligible compared to the uncertainties of both approaches. Also the accuracy of predictions improves as expected with the quality of the reduction method, with the exception of *V*(⟨*r*⟩) from the calculations of refs 36, 38, and 65 . The reason here is that the 12D CCpol-8sf PES used in these calculations reduces at ⟨*r*⟩ to the very accurate 6D CCpol-8s surface, whereas the corrections for monomers’ deformations are computed from much less accurate SAPT-5sf surface of ref 34.

There were also a number of papers reporting both 4D and 6D diatom–diatom scattering calculations.^18,19,43–45^ One of the most studied such systems is H_2_–CO. Calculations of ref 18 have shown that cross sections for excitations of CO by H_2_ follow a similar accuracy progression as in the case of spectra, with 4D ⟨*V*⟩ and 6D cross sections essentially overlapping. Furthermore, the differences between all studied approaches are smaller than the experimental error bars. In the study of ref 45, the cross sections for rotational excitation of HCl in collisions with *para*-H_2_ computed using the 4D ⟨*V*⟩ PES are indistinguishable on the scale of the plot from 6D results, while the ⟨*r*⟩ cross sections are markedly different and *r*_e_ ones almost qualitatively different. These calculations were not compared to experiment. Recently Zhang et al.^68^ published the first scattering calculations for diatom-triatom (H_2_–H_2_O) in full dimensionality (9D). They also computed the cross sections in 8D, with rigid H_2_ at the *r*_e_ geometry. According to the authors, their results suggest that “rigid-rotor treatment to H_2_ is good enough to describe inelastic scattering between H_2_O and H_2_, where H_2_ does not undergo a vibrational transition”. It almost certainly would have been still better had the authors used the vibrationally averaged PES. Note that since no comparisons with experiment were made in ref 68, the relevance of the discrepancies between 9D and 8D approaches relative to errors with respect to experiment cannot be evaluated.

## Conclusions

We have investigated here the importance of including monomer flexibility effects in calculations of rovibrational spectra, using as an example the H_2_–CO dimer. Applying the recently published^52^ full-dimensional (6D) PES, we have developed five types of reduced-dimensionality (4D) surfaces: two with fixed *r*_*i*_, two with Taylor’s expansion of *V* averaged over monomer vibrations, and one with exact averaging. The rovibrational energy levels of *para*- and *ortho*-H_2_–CO from the 6D and from the five 4D approaches have been compared with the experimental levels. While the most popular 4D approximation, *V*(*r*_1*e*_, *r*_2*e*_), gives errors with respect to experiment that are an order of magnitude larger than those given by the 6D approach, all three *V*-averaging approximations perform as well as 6D, in fact, have all slightly lower errors than 6D. Actually, the differences between the latter approaches are so small that this comparison may be effected by uncertainties of experimental results. This leads to averaged-*V* 4D spectra being practically indistinguishable from the 6D spectrum, with a much smaller computational effort involved. While we were expecting these two types of predictions to be similar, such closeness is surprising. The excellent performance of the Taylor expansion methods is particularly important since not only the costs of the nuclear dynamics calculations can be significantly reduced by going from 6D to 4D, but also in order to get ⟨*V*⟩_00_^TE^ there is no need to develop the full-dimensional interaction energy surface (while it is needed for ⟨*V*⟩_00_) and the derivatives involved can be computed at a lower level of theory than the leading term^55,69^ without any noticeable loss of accuracy. This feature makes the generation of an averaged surface only modestly more expensive than the generation of the corresponding surface based on fixed monomer geometries.

To address the question whether good performance of reduced dimensionality approximations based on averaging (rigorous and TE) found for H_2_–CO can be expected for other dimers, particularly dimers with stronger interactions, we examined literature calculations that used both full and reduced dimensionality approaches (see Table SX **in SI**) and found that for Ar–HF and (H_2_O)_2_ the differences between the two approaches are larger than for H_2_–CO. These findings could be expected from analysis based on perturbation theory, with the intermolecular interaction treated as a perturbation of the vibrational motion within the molecules.^46^ Since in the averaging leading to ⟨*V*⟩, ⟨*V*⟩^TE^, and ⟨*V*⟩^TE3^ we use the wave functions of the isolated (unperturbed) molecules, we neglect the effects of this perturbation. If the intermolecular interactions are stronger, such effects will be stronger, and therefore such reduced-dimensionality approximations will be less accurate. However, it does not imply that the reduced dimensionality approximations will not perform well for such systems. The reason is that in most cases dimers with strong interactions are also larger than H_2_–CO and the best achievable accuracy of full-D PESs of such systems will be much smaller than that of the present PES. One can see this by comparing errors relative to experiment of our 6D calculations for H_2_–CO with analogous errors for the HF dimer calculations of ref 70: about 0.01 cm^–1^ (RMSE of energy levels) versus 1.40 cm^–1^ (RMSE of transition energies for the ν_1_ + ν_2_ = 0 manifold computed using only the results from recent accurate measurements of ref 71). Since both PESs and the nuclear dynamics calculations represent the limits of what can be done at the present time, the factor over 100 difference in these errors illustrates our point. The differences between the best 4D and 6D approaches are of the order of 0.001 cm^–1^ for H_2_–CO. Even if these differences were 2 orders of magnitude larger for HF–HF, i.e., amount to 0.1 cm^–1^, so that RMSE in 4D would be 1.30 or 1.50 cm^–1^, an irrelevant difference. In fact, it is possible that 4D calculations can reach a better agreement with experiment than 6D ones since with smaller number of grid points needed, one can afford a more advanced *ab initio* method and a better basis set. This hypothesis needs to be tested.

If it is confirmed, our method opens avenues for computational exploration of matter that would not be possible with full-dimensional approach due to computational costs. The ⟨*V*⟩ approach can currently be applied to develop PESs for dimers consisting of two 5-atomic monomers (24 DoFs, restricted by costs of computing training data set), and even larger dimers in the TE approximation. Such PESs are at the most 6-dimensional, so they can be used in the near-exact 6D quantum nuclear dynamics calculations. Thus, the reduction of dimensionality opens doors for doubling the current range of rigorous investigations of matter from about 12 (9) DoFs treated in full dimensionality in the case of spectra (scattering) to 24 actual DoFs reduced to at most 6 DoFs.

The increased predictive power of nuclear dynamics based on reduced dimensionality aiFFs may be of importance in several fields of physics, chemistry, and engineering. First, such aiFFs can be used in any MD simulations of soft condensed matter properties. The next application are spectra of dimers (vibration-rotation-tunneling spectra were measured for dimers as large as the benzene dimer^72^) and scattering calculations, including those relevant for cold and ultracold physics involving polyatomic molecules.^21^ Spectra of a large number of polyatomic molecules have been observed in space, in particular in cold interstellar clouds, and to interpret such spectra, one needs to know their rotational state distribution due to collisional excitations and de-excitations by He and H_2_, which can be inferred from scattering calculations such as those performed for methyl formate, the largest “prebiotic” molecules observed in space.^73,74^ Finally, for crystals built from small and medium size rigid monomers, computationally predicted structures can be very close to experiment^1,75^ and such predictions may be sensitive to monomer’s geometry. Our approach will not apply, however, to crystals built of molecules with soft degrees of freedom, such a rotatable bonds, since such molecules may be significantly deformed in crystals.

## Methods

### Potential Energy Surfaces

The 6D potential energy surface *V* ^52^ used in the present work was fitted to energies computed using the coupled cluster (CC) method^76,77^ with single, double, triple, and noniterative quadruple excitations, CCSDT­(Q), assuming the Born–Oppenheimer (BO) approximation and neglecting relativistic effects. Large basis sets calculations were followed by extrapolations to the complete basis set (CBS) limit. The uncertainty of the computed interaction energies relative to the exact solutions of Schrödinger’s equation in the relativistic adiabatic approximation was estimated in ref 56 to amount to 0.6 cm^–1^ in the region of the van der Waals minimum (or 0.8%). Also, a very large set of 34,559 grid points was used. The results were fitted by a functional form to within 0.02 cm^–1^ for points with negative interaction energies (the region relevant for spectral calculations). Thus, the overall uncertainty of our PES is determined by uncertainties of *ab initio* calculations, i.e., it is below 1 cm^–1^. This uncertainty may seem large compared to the RMSEs of energy levels with respect to experiment shown in Figure 1, which in 6D treatment are 0.0052 and 0.0127 cm^–1^ for the *para* and *ortho* cases, respectively. What makes such small errors possible are significant cancellations of energy errors in calculations of energy levels which are given relatively to the ground-state energy. Further information about the *V* surface is given in SI. To perform the 6D calculations, we used the total PES defined as *U*_23_ = *V*_23_ + *V*_HH_ + *V*_CO_, where *V*_HH_ ^78^ and *V*_CO_ ^79,80^ are the monomer potentials.

Two of five 4D surfaces tested in our work, *V*(*r*_1*e*_, *r*_2*e*_) and *V*(⟨*r*_1_⟩_*v*_1__, ⟨*r*_2_⟩_*v*_2__), are straightforward to obtain from the 6D *V*(*R*, θ_1_, θ_2_, ϕ, *r*_1_, *r*_2_), with the values of *r*_1_ and *r*_2_ set to *r*_1*e*_ = 1.4011, *r*_2*e*_ = 2.1322, ⟨*r*_1_⟩_0_ = 1.44835, and ⟨*r*_2_⟩_0_ = 2.13993 bohr, respectively (computed by us from *V*_HH_ and *V*_CO_). The ⟨*V*⟩_*v*_1_ *v*_2__^TE^, ⟨*V*⟩_*v*_1_ *v*_2__^TE3^, and ⟨*V*⟩_*v*_1_ *v*_2__ surfaces were calculated from V in the following way. The ⟨*V*⟩_*v*_1_ *v*_2__^TE^ surface is obtained by first replacing the PES by its Taylor expansion (TE) in *r*_1_ and *r*_2_ up to second-order terms and then averaging over monomer vibrations^54–56^

1 $$\langle V\rangle_{v_{1}v_{2}}^{\mathrm{TE}}\left(X\right)=f_{00}^{c}\left(X\right)+f_{10}^{c}\left(X\right)\left(\langle r_{1}\rangle_{v_{1}}-r_{1c}\right)+f_{01}^{c}\left(X\right)\left(\langle r_{2}\rangle_{v_{2}}-r_{2c}\right)+f_{11}^{c}\left(X\right)\left(\langle r_{1}\rangle_{v_{1}}-r_{1c}\right)\left(\langle r_{2}\rangle_{v_{2}}-r_{2c}\right)+\frac{1}{2}f_{20}^{c}\left(X\right)\left(\langle r_{1}^{2}\rangle_{v_{1}}-2r_{1c}\langle r_{1}\rangle_{v_{1}}+r_{1c}^{2}\right)+\frac{1}{2}f_{02}^{c}\left(X\right)\left(\langle r_{2}^{2}\rangle_{v_{2}}-2r_{2c}\langle r_{2}\rangle_{v_{2}}+r_{2c}^{2}\right)$$

where ***X*** is the set of intermonomer coordinates, *f*_*ij*_^*c*^ is the *i*th (*j*th) derivative of *V* with respect to *r*_1_ (*r*_2_) at point (*r*_1*c*_, *r*_2*c*_), the averages ⟨*r*_1_^*n*^⟩_*v*_*i*__ were computed by us using the wave functions of isolated H_2_ with *v*_1_ = 0 and *j*_1_ = 0 or 1 and those of isolated CO with *v*_2_ = 0 or 1 and *j*_2_ = 0.^78–80^ The ⟨*V*⟩_*v*_1_ *v*_2__^TE3^ surface can be obtained by an augmentation of ⟨*V*⟩_*v*_1_ *v*_2__^TE^ with the averaged third-order Taylor expansion terms (see **Sec. SIV in** SI). The ⟨*V*⟩_*v*_1_ *v*_2__ surface is the directly averaged *V*. In comparison to refs 55, 56 , where the derivatives needed for TE were computed on a 4D grid and then a 4D PES was fitted, here we calculated ⟨*V*⟩_00_^TE^ and ⟨*V*⟩_00_^TE3^ on-the-fly while running nuclear dynamics calculations using the BOUND program.^57,58^ The averaging

$${\left\langle V\right\rangle }_{v_{1}v_{2}}=\left\langle \chi_{v_{1}}\chi_{v_{2}}\left|V\right|\;\chi_{v_{1}}\chi_{v_{2}}\right\rangle$$ 2

where χ_*v*_1__(*r*_1_) and χ_*v*_2__(*r*_2_) are calculated for the isolated monomers, was also performed on-the-fly, with precalculated χ_*v*_1__(*r*_1_) and χ_*v*_2__(*r*_2_) obtained from the TRIATOM program.^81^ On-the-fly procedures allowed us to avoid potential inaccuracies introduced by the 4D fitting. Notice that while here ⟨*V*⟩_*v*_1_ *v*_2__, ⟨*V*⟩_*v*_1_ *v*_2__^TE^, and ⟨*V*⟩_*v*_1_ *v*_2__^TE3^ were all obtained from 6D *V*, in future applications the latter two surfaces can be constructed without knowledge of the full-dimensional surface.

The use of truncated Taylor expansion to represent intramonomer PESs is common in computational molecular science, in particular the harmonic and third-order anharmonic approximations are standard approaches in analyzing molecular vibrations. One may point out that the truncated Taylor expansion method used by us is different from the applications of this expansion to molecular clusters in literature. For example, Le Roy and van Kranendonk used it for H_2_ in ref 48 (see also ref 82), but it was just the assumed form of their empirical PES, with the coefficients obtained by fitting to experimental spectra. Similarly, truncated Taylor expansions were used in ref 83 for He–HF and in ref 50 for H_2_–H_2_O to represent the full-dimensional PES and the coefficients of the resulting polynomials were parameters of the fit to *ab initio* data (this form also makes averaging over monomer vibrations straightforward). In our Taylor’s expansion methods, the quantities in front of powers of variables are not parameters of the fit, but partial derivatives of the surface computed from first principles. In standard applications, these derivatives are computed on a grid for each point in intermonomer coordinate space (and, as already mentioned, can be computed at a lower level of theory than the 4D base PES, see ref 55). In the present case, since we had an accurate 6D PES available, the derivatives were computed using this surface.

### 6D Nuclear Dynamics

Due to very high precision needed to compare 4D with 6D spectra, we have performed extensive tests of the methodology of the 6D rovibrational calculations from the point of view of their uncertainties. This resulted in a revised set of energy levels compared to the calculations of ref 52. To perform the 6D rovibrational calculations, a body-fixed frame was used, defined such that its *z*-axis is along the vector ***R*** connecting the COMs of the monomers and its *x*-axis is along ***R*** × (***r***_1_ × ***R***). The 6D rovibrational levels were calculated using a nearly exact variational method that expands the rovibrational wave functions in a product basis set with discrete variable representation (DVR) functions for the stretch coordinates and spherical harmonic type functions for the bending coordinates. A symmetry-adapted Lanczos (SAL) method^84^ is used to solve the resulting eigenvalue problem. The complete approach is denoted as DSL^85,86^ (DVR + spherical + Lanczos). The calculations were carried out with the RV4 code.^87^ The DSL method is described in details in refs 88 and 89 . Each basis function in the DSL method is of the form

3 $$f_{\alpha_{0}}\left(r_{0}\right)\;f_{\alpha_{1}}\left(r_{1}\right)\;f_{\alpha_{2}}\left(r_{2}\right)u_{j_{1}j_{2}m_{2}K}^{JMp}\left(\theta_{1},\theta_{2},\phi ;\alpha ,\beta ,\gamma \right)$$

where *f*_α_*i*__(*r*_*i*_) is a DVR function for the stretching coordinate *r*_*i*_ (*i* = 0, 1, 2) with *r*_0_ = *R*, *u*_*j*_1_ *j*_2_ *m*_2_ *K*_^*JMp*^ is a parity-adapted bend-rotation function, and α, β, γ are Euler angles specifying the orientation of the body-fixed frame in the space-fixed frame. The rotational quantum numbers *J*, *j*_1_, *j*_2_ are for the overall rotations and these of monomers, and *K*, *m*_1_, *m*_2_ are quantum numbers of the operators that are the projections of the angular momentum operators *Ĵ*, *ĵ*_1_, *ĵ*_2_ on the body-fixed frame. However, *m*_1_ is omitted in the subscript of *u*_*j*_1_ *j*_2_ *m*_2_ *K*_^JMp^ because it is related to *K* via *K* = *m*_1_ + *m*_2_. The functions *u*_*j*_1_ *j*_2_ *m*_2_ *K*_^*JMp*^(θ_1_, θ_2_, ϕ; α, β, γ) are linear combinations of two products of an associated Legendre function of θ_1_, a spherical harmonic function of (θ_2_, ϕ), and a Wigner function of the Euler angles (α, β, γ), with the parity indicated by *p*. In the DSL method, potential energy integrals are computed using a Gauss quadrature. Eigenvalues and eigenvectors are determined with the SAL algorithm^84,90^ implemented with the full *G*_4_ permutation-inversion group.

The basis functions parameters are chosen by extensive convergence tests against a large benchmark basis (basis I in **Table SXI in** SI). The *N*_*r*_0__ = 80 sine DVR basis in the range [4.0, 50.0] bohr is used for the *r*_0_ coordinate. The *r*_0_ basis is not optimized to avoid any bias. The large maximum *r*_0_ value ensures the convergence of the bound states near dissociation threshold. Potential optimized DVR (PODVR) bases^91,92^ are used for *r*_1_ and *r*_2_ coordinates. The reference potentials for the PODVRs are taken to be the potentials of free H_2_ and CO. For the angular basis, we use max­(*j*_1_, *m*_1_) = 3 (H_2_) and max­(*j*_2_, *m*_2_) = 10 (CO). The numbers of quadrature points are (*N*_θ_1__, *N*_θ_2__, *N*_ϕ_) = (6, 11, 22), where Gauss-Legendre points are used for θ_1_ and θ_2_ and Fourier points are used for ϕ. All parameters used in the calculations are given in Table SXI. Basis II with *N*_*r*_1__ = 3 (H_2_) and *N*_*r*_2__ = 5 (CO) was used in ref 52. It was estimated there that all the bound states are converged to within 0.0001 cm^–1^ for *para-*H_2_–CO except for a few bound states near the dissociation limit and the resonance states. This accuracy estimate was based on comparing levels computed with *N*_*r*_1__ = 3 and *N*_*r*_1__ = 5 basis (basis III in Table SXI). In the present work, we find that for *ortho-*H_2_–CO levels, to maintain the 0.0001 cm^–1^ accuracy, the *N*_*r*_1__ = 5 basis is necessary because the intramolecular and intermolecular couplings, while weak for the *para*-H_2_–CO levels up to about 21 cm^–1^ above the ground state, become stronger for the *ortho-*H_2_–CO levels that start from about 117 cm^–1^. The energy of the *ortho*-H_2_–CO ground state, denoted oGS, increases by a significant 0.0823 cm^–1^ (from 117.1292 to 117.2115 cm^–1^) when *N*_*r*_1__ = 3 is increased to *N*_*r*_1__ = 5. However, experimental levels of *ortho*-H_2_–CO were measured relative to the *ortho*-H_2_–CO ground state because transitions between *para* and *ortho* states are forbidden. Relative to the oGS, the difference between *ortho*-H_2_–CO levels computed with *N*_*r*_1__ = 3 basis and *N*_*r*_1__ = 5 basis is much smaller, only 0.0001 cm^–1^ for levels up to about 14 cm^–1^ and 0.0007 cm^–1^ for all the bound states. In this work, we therefore used the larger *N*_*r*_1__ = 5 basis to compute both the *para*-H_2_–CO and *ortho-*H_2_–CO levels. This ensures the 0.0001 cm^–1^ accuracy.

There are three bound states for *ortho*-H_2_–CO (*v*_2_ = 0) that can be classified as excited in the van der Waals stretch, i.e., (*J*, *P*, *n*_*J,P*_) = (0, *f*, 3), (1, *f*, 8), and (1, *e*, 11) (for labeling of the states, see Sec. SV in SI). For these states, much larger values of the intermolecular distance *r*_0_ are probed, and to converge their energies to 0.0001 cm^–1^, we had to increase *r*_0max_ to 100 bohr. Also, *N*_*r*_0__ was proportionally increased to 170. This test was done on 4D V_12_ PES, using a nonoptimized basis for *r*_0_. Our numerical tests have shown that for the most difficult to converge (0,f,3) state, the inaccuracy is less than 0.0004 cm^–1^.

### 4D Nuclear Dynamics

The 4D nuclear dynamics calculations were performed for the bound states with all 4D PESs using the BOUND package.^57,58^ BOUND applies the coupled-channel method within the space-fixed coordinate formalism. These calculations are similar to those of refs 49, 55, 56, and 93 . The parameters controlling the convergence of the calculations were the same as in refs 55 and 56 except that the maximum value of *j*_2_, defining the size of the angular basis set on CO, was set to 10, the maximum order of spherical harmonics in the angular coordinates of H_2_ and CO used by BOUND to project out the interaction potential was set to 12 in both cases, and the propagation step size was 0.03 Å. With these parameters, numerical uncertainties of rovibrational energies are below 0.0001 cm^–1^. The rotational constants used: *B*_0_^1^ = 59.3383 cm^–1^ (for H_2_), *B*_0_^1^ = 29.9145 cm^–1^ (for D_2_), and *B*_0_^2^ = 1.9226 cm^–1^ (for CO) were computed by us from *V*_HH_ ^78^ and *V*_CO_.^79,80^ They were obtained from the formula $B_{0}^{i}=\frac{1}{2\mu_{i}}\left\langle \chi_{v_{i}}\left|\frac{1}{r_{i}^{2}}\right|\chi_{v_{i}}\right\rangle$ , where μ_*i*_ are the reduced masses.

## Supplementary Material



## Notes

Additional information about the coordinates, the interaction energy surface, the construction of approximate surfaces, and tables with data used for plots in the article and with basis set parameters for the 6D calculations (PDF)
