# Supplementary material for: Importance of Monomer-Flexibility Effects for Spectra of Molecular Clusters
Source: J Phys Chem Lett. 2026 Apr 20;17(17):4982–91. doi: 10.1021/acs.jpclett.6c00742 (PMC13308992; doi:10.1021/acs.jpclett.6c00742)
Supplement: Supplementary file 1 [file jz6c00742_si_001.pdf]

Supporting Information for

Importance of Monomer-flexibility Effects for Spectra of Molecular Clusters

Marcin Stachowiak,[†] Ewelina Grabowska,[†] Xiao-Gang Wang,[‡] Tucker Carrington Jr.,[‡] Krzysztof Szalewicz,[§] and Piotr Jankowski[*,†]

† *Faculty of Chemistry, Nicolaus Copernicus University in Toruń, Gagarina 7, 87-100 Toruń, Poland*

‡ *Chemistry Department, Queen's University, Kingston, Ontario K7L 3N6, Canada*

§ *Department of Physics and Astronomy, University of Delaware, Newark, DE 19716, USA*

∗ E-mail: piotr.jankowski@umk.pl

SI. EXTENDED DISCUSSION OF LITERATURE

If an aiFF depends on all degrees of freedom (DoFs), the size of molecules to which the aiFF approach can be applied is a severe limitation of this approach. Currently, the number of DoFs that can be treated at high accuracy in *ab initio* electronic structure calculations is 20 or so (e.g., water trimer). While aiFFs with 20 DoFs can be applied in MD simulations of matter using classical or quasi-classical mechanics, the current applicability of accurate quantum nuclear dynamic methods is limited to 12 DoFs for rovibrational spectraand 9 DoFs for quantum scattering calculations [1]. The first calculations of rovibrational spectra in 6D, were performed in early 1990s [2–6] using the HF dimer aiFF of Ref. [7], a system that is also of current interest [8–10]. Spectra from a 12D aiFF water dimer PES were computed in 2006 [11] using a 6D+6D nuclear dynamics approach of Ref. [12]. The first full 12D nuclear dynamics calculations were performed only in 2018 [13] using the aiFF of Ref. [14] and this dimer is also of continued interest [15–17]. The first 6D scattering calculation were performed in 2002 [18] for the H_2 dimer on the aiFF of Ref. [19]. Similar 6D calculation were later performed for a number of diatom-diatom dimers [20–26]. One may point out that empFFs are almost always all-dimensional (and classical dynamics can be performed for millions DoFs). However, this is at the cost of neglecting essentially all couplings between DoFs. This leads to errors of energies predicted by empFFs to be several kcal/mol, while for calculations of cluster spectra that resemble experimental ones one needs to reduce the errors to about 1 cm^{-1} or 0.003 kcal/mol, three orders of magnitude difference.

SII. JACOBI COORDINATES

The Jacobi coordinates that have been used to describe the geometry of the H_2–CO complex are as follows (see Fig. S1): the distance between the centers of masses (COMs) of the monomers, $R = |\boldsymbol{R}|$, three angles θ_1 ($\boldsymbol{R}$-$\boldsymbol{r_1}$ angle), θ_2 ($\boldsymbol{R}$-$\boldsymbol{r_2}$ angle), and ϕ (the dihedral angle), and the intramonomer distances $r_1 = |\boldsymbol{r_1}|$ and $r_2 = |\boldsymbol{r_2}|$.

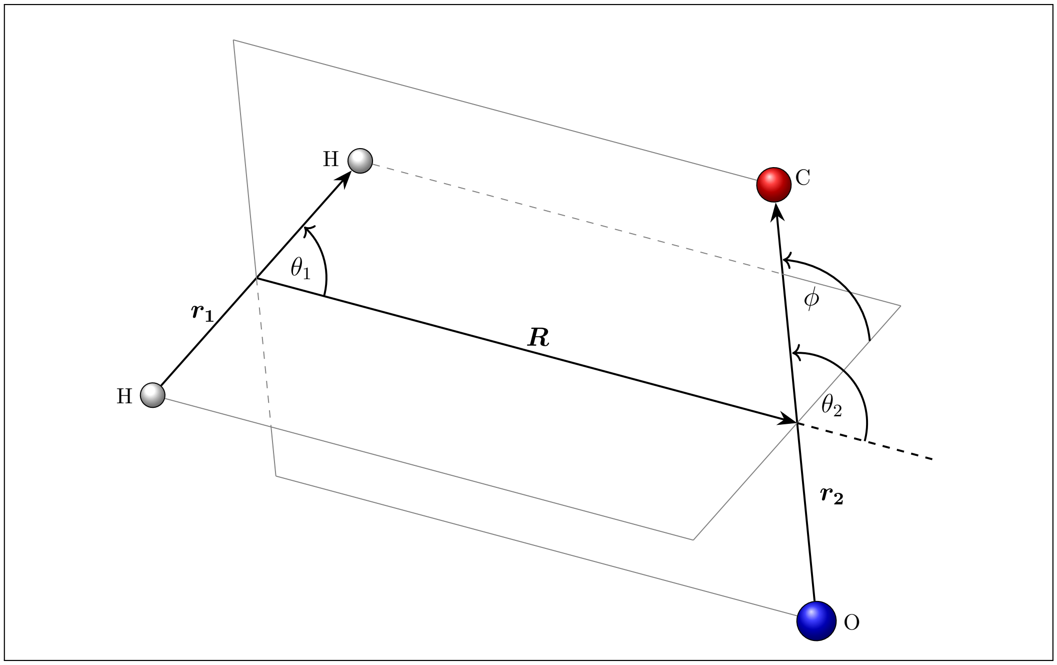

FIG. S1. Definition of the Jacobi coordinates for the H_2–CO complex. The $\boldsymbol{r_1}$ ($\boldsymbol{r_2}$) vector connects the H nuclei (the O and C nuclei), with H1 at the beginning of the vector, whereas the $\boldsymbol{R}$ vector connects the center of masses (COM) of H_2 with the COM of CO.

SIII. 6D INTERACTION ENERGY SURFACE

A. Calculations of interaction energies

We will use the 6D analytic PES developed in Ref. [27]. The 'vertical' *ab initio* training interaction energies were computed using the supermolecular approach and were counterpoise corrected. The aug-cc-pVXZ basis sets [28] were used. For distances between the centers of mass (COM) $R \leq 13.5$ bohr, we applied the CCSDT(Q) method with an $X =$ (Q5) basis, where parentheses denote CBS extrapolations, at CCSD(T) level and $X =$ D basis with frozen core approximations at the T(Q) level. For $R > 13.5$ bohr, we used bases with cardinal numbers lowered by one and omitted the T(Q) term. The uncertainty of the interaction energies computed at the highest level of theory was estimated to be equal to 0.6 cm^{-1} in the van der Waals minimum region, which amounts to 0.8% of the total interaction energy at the global minimum.

B. Fit 6D

Different functional forms were used to fit interaction energies in the short-range and in the asymptotic regions, denoted as $V_{\rm sh}$ and $V_{\rm as}$, respectively. The training set for $V_{\rm sh}$ included 34,559 points in the range $4.5 \leq R \leq 12$ bohr and the bond lengths of the monomers varying in the range 0.95 to 2.05 bohr for H_2 and 1.90 to 2.45 bohr for CO. For $V_{\rm as}$, a set of 20,972 points was used with $10 \leq R \leq 20$ bohr. Note that the points (9,307 of them) in the region $10 \leq R \leq 12$ were used in both fits. Also note that the interaction energies in the $V_{\rm as}$ training set for grid points with $10 \leq R \leq 13.5$ bohr were computed at the higher level of theory, while only those with $R > 13.5$ bohr were computed at the lower level.

To represent the short-range component, $V_{\rm sh}$, we have employed an idea similar to that used earlier by Fernandez *et al.* [29] or by the Bowman group [30] to use a linear expansion in the basis of multivariate polynomials of the internuclear distances q_i, which are invariant with respect to any permutation of the equivalent nuclei of the complex. In the case of H_2-CO, there are only two equivalent nuclei. There are six internuclear distances, q_i, defined as follows: $q_1 = d_{\rm H_AH_B}$, $q_2 = d_{\rm CO}$, $q_3 = d_{\rm H_AO}$, $q_4 = d_{\rm H_BO}$, $q_5 = d_{\rm H_AC}$, and $q_6 = d_{\rm H_BC}$. Thus, q_1 and q_2 are the intramolecular distances. To find the form of the permutation-invariant polynomials, we have developed a computer program that generates them using the basic properties of permutations for a given type of interacting molecules. It should be emphasized here that each of the polynomials is invariant, thus we can pick a set of them for further considerations without any restrictions. One has to find a balance between the number of the polynomials taken into account and the quality of the fit.

In fact, similarly to Bowman's group approach [30], our polynomials do not depend directly on the q_i variables but on the functions $f = e^{-\alpha q_i}$. Such form is analogous to using the Morse-like functions of q_i instead of the bare variables q_i. We have noticed that the overall performance of the fit significantly depends on the α exponent in $e^{-\alpha q_i}$. Moreover, we have found that the efficiency of the fit increases if, instead of using high-degree polynomials, one uses functions f with several distinct exponents, and lower-order polynomials are built out of them. Since in principle we can use any combination of the permutation-invariant polynomials to build the analytic representation of the surface, there is a large variety of possible choices. One has to perform some systematic tests to find an optimal form for the H_2-CO case that fulfills two conditions: a very accurate fit to the training set and almost as

accurate predictions for a test set of energies. To achieve this goal, we used four adjustable exponents: one for the intramolecular functions $f_1 = e^{-\alpha_a q_1}$ and $f_2 = e^{-\alpha_a q_2}$, and three others defining three sets of functions: $f_i = e^{-\alpha_b q_i}$, where $i = 3, \ldots, 6$, $f_i = e^{-\alpha_c q_{(i-4)}}$, $i = 7, \ldots, 10$, and, finally, $f_i = e^{-\alpha_d q_{(i-8)}}$, $i = 11, \ldots, 14$. As a consequence, $V_{\rm sh}$ is a function of six internuclear distances q_i, but through fourteen variables f_i. Next, we have introduced seven classes of permutation-invariant polynomials depending on the type of variables employed. The first one comprises only the f_1 and f_2 variables. The second, third, and fourth classes are built of the polynomials of f_i for $i = 3, \ldots, 6$, f_i for $i = 7, \ldots, 10$, and $i = 11, \ldots, 14$, respectively. The fifth class contains the polynomials of f_i with the indices $i = 1, \ldots, 6$, the seventh class contains the polynomials of f_i with the indices $i = 1, 2, 7, \ldots, 10$, whereas the seventh class with the indices $i = 1, 2, 11, \ldots, 14$. Thus, in the present model, there is no mixing of the f_i functions belonging to three distinct subsets defined by $i = 3, \ldots, 6$, $i = 7, \ldots, 10$, and $i = 11, \ldots, 14$, in any of the permutation-invariant polynomials. After the suitable polynomials are formed, for each K-class of polynomials, $K = 1, \ldots, 7$, we define V_K as a linear combination of the polynomials $w_{K,i}$ belonging to this class:

$$V_K = \sum_i c_{K,i} w_{K,i}, \tag{1}$$

where the summation in 1 runs over all chosen polynomials. Then, the short-range potential $V_{\rm sh}$ can be written in the form

$$V_{\rm sh} = \sum_{K=1}^{7} V_K. \tag{2}$$

Let us also assume that zero-order polynomial, just 1, belongs to the class V_1. The highest degree of the polynomials used in the present form of the fit is equal to 6, but to further restrict the flexibility of the fit, we have introduced a constraint that the power of any single variable f_i is 3 or less. For instance, the terms $f_1^3 f_2^3$ are included, but $f_1^4 f_2^2$ are not. The final form of $V_{\rm sh}$ has 1198 linear parameters $c_{K,i}$ and four nonlinear parameters α_a, α_b, α_c, and α_d. The total number of linear coefficients comes from one zero-order coefficient, 15 coefficients of the terms composed of only f_1 and f_2 functions, and the three sets of 394 coefficients corresponding to the terms defined by the f_i variables related to α_b, α_c, and α_d.

The long-range component $V_{\rm as}$ has the same form as in the V_{15} surface [22, 31], i.e., it is an asymptotic expansion in powers of $1/R^n$, starting from $n = 4$, multiplied by appropriate angular factors. The functions $V_{\rm sh}$ and $V_{\rm as}$ were both contributing in the region $10 \le R \le 12$

bohr, mixed using a smooth switching function. The resulting 6D intermolecular interaction energy surface is dubbed V_{23}. It has an RMS errors of 0.340 cm^{-1} and 0.021 cm^{-1} for all data and those with negative interaction energies, respectively.

SIV. APPROXIMATIONS BASED ON THE TAYLOR EXPANSION OF THE INTERACTION ENERGY

The full-dimensional energy surface $V(\boldsymbol{X}, r_1, r_2)$, $\boldsymbol{X} = \{R, \theta_1, \theta_2, \phi\}$, can be approximated by the $\tilde{V}(\boldsymbol{X}, r_1, r_2)$ surface obtained as the Taylor expansion of V with respect to r_1 and r_2 around some reference distances r_{1c} and r_{2c}, respectively, and truncated at the quadratic terms

$$\begin{aligned}\tilde{V}(\boldsymbol{X}, r_1, r_2) = {} & f^c_{00}(\boldsymbol{X}) + f^c_{10}(\boldsymbol{X})(r_1 - r_{1c}) + f^c_{01}(\boldsymbol{X})(r_2 - r_{2c}) \\ & + f^c_{11}(\boldsymbol{X})(r_1 - r_{1c})(r_2 - r_{2c}) \\ & + \frac{1}{2} f^c_{20}(\boldsymbol{X})(r_1 - r_{1c})^2 + \frac{1}{2} f^c_{02}(\boldsymbol{X})(r_2 - r_{2c})^2,\end{aligned} \tag{3}$$

where $f^c_{ij}(\boldsymbol{X})$ are the $(i+j)$-th numerical partial derivatives of V, i-th (j-th) with respect to r_1 (r_2), evaluated at the reference intramolecular separations r_{1c} and r_{2c}, respectively, for a given values of the intermolecular coordinates $\boldsymbol{X}$. The vibrationally-averaged Taylor-expanded interaction energy $\langle \tilde{V} \rangle_{v_1 v_2}$ is defined as

$$\begin{aligned}\langle \tilde{V} \rangle_{v_1 v_2}(\boldsymbol{X}) = {} & f^c_{00}(\boldsymbol{X}) + f^c_{10}(\boldsymbol{X})(\langle r_1 \rangle_{v_1} - r_{1c}) + f^c_{01}(\boldsymbol{X})(\langle r_2 \rangle_{v_2} - r_{2c}) \\ & + f^c_{11}(\boldsymbol{X})(\langle r_1 \rangle_{v_1} - r_{1c})(\langle r_2 \rangle_{v_2} - r_{2c}) \\ & + \frac{1}{2} f^c_{20}(\boldsymbol{X})(\langle r_1^2 \rangle_{v_1} - 2r_{1c}\langle r_1 \rangle_{v_1} + r_{1c}^2) + \frac{1}{2} f^c_{02}(\boldsymbol{X})(\langle r_2^2 \rangle_{v_2} - 2r_{2c}\langle r_2 \rangle_{v_2} + r_{2c}^2),\end{aligned} \tag{4}$$

where the averages of powers of r_1 and of r_2, $\langle r_1^n \rangle_{v_1}$ and $\langle r_2^n \rangle_{v_2}$, were computed with the wave functions of the $v_1 = 0$ and $j_1 = 0$ or 1 rovibrational state of the isolated H_2 and of the $v_2 = 0$ or 1 and $j_2 = 0$ rovibrational states of the isolated CO. Since in the Taylor-expansion approximation the averaging is not dependent on $\boldsymbol{X}$, the vibrationally averaged values $\langle r_1^n \rangle_{v_1}$ and $\langle r_2^n \rangle_{v_2}$ were precomputed. The reference separations are set to $r_{1c} = 1.44835$ bohr and $r_{2c} = 2.13993$ bohr.

In our work, we use $\langle V \rangle^{\mathrm{TE}}_{v_1 v_2}$ to denote the surface $\tilde{V}$ obtained by averaging over the vibrations of the monomers, the second-order Taylor expansion of the full-dimensional interaction energy surface V. The rovibrational energies calculated from that surface are compared to

their counterparts obtained from $\langle V\rangle_{v_1v_2}$. To make further comparison, we can introduce the $\langle V\rangle^{\mathrm{TE3}}_{v_1v_2}$ surface, obtained by averaging the third-order Taylor expansion of V. This expansion can be defined as

$$\begin{aligned}\bar{V}(\boldsymbol{X},r_1,r_2) = \tilde{V}(\boldsymbol{X},r_1,r_2)&\\ +\frac{1}{6}f^c_{30}(\boldsymbol{X})(r_1-r_{1c})^3+\frac{1}{2}f^c_{21}(\boldsymbol{X})(r_1-r_{1c})^2(r_2-r_{2c})&\\ +\frac{1}{2}f^c_{12}(\boldsymbol{X})(r_1-r_{1c})(r_2-r_{2c})^2+\frac{1}{6}f^c_{03}(\boldsymbol{X})(r_2-r_{2c})^3,&\end{aligned} \tag{5}$$

where $\tilde{V}(\boldsymbol{X},r_1,r_2)$ is defined by Eq. 3, and $f^c_{ij}(\boldsymbol{X})$ are the i-th and j-th numerical partial derivatives of V defined above. One can average $\bar{V}(\boldsymbol{X},r_1,r_2)$ over the vibrational modes v_1 and v_2 of the molecules, to obtain

$$\begin{aligned}\langle\bar{V}\rangle_{v_1v_2}(\boldsymbol{X}) = \langle\tilde{V}\rangle_{v_1v_2}(\boldsymbol{X})&\\ +\frac{1}{6}f^c_{30}(\boldsymbol{X})(\langle r_1^3\rangle_{v_1}-3r_{1c}\langle r_1^2\rangle_{v_1}+3r_{1c}^2\langle r_1\rangle_{v_1}-r_{1c}^3)&\\ +\frac{1}{2}f^c_{21}(\boldsymbol{X})(\langle r_1^2\rangle_{v_1}-2r_{1c}\langle r_1\rangle_{v_1}+r_{1c}^2)(\langle r_2\rangle_{v_2}-r_{2c})&\\ +\frac{1}{2}f^c_{12}(\boldsymbol{X})(\langle r_1\rangle_{v_1}-r_{1c})(\langle r_2^2\rangle_{v_2}-2r_{2c}\langle r_2\rangle_{v_2}+r_{2c}^2)&\\ +\frac{1}{6}f^c_{03}(\boldsymbol{X})(\langle r_2^3\rangle_{v_2}-3r_{2c}\langle r_2^2\rangle_{v_2}+3r_{2c}^2\langle r_2\rangle_{v_2}-r_{2c}^3),&\end{aligned} \tag{6}$$

where the averaged values of powers of r_1 and of r_2, $\langle r_1^n\rangle_{v_1}$ and $\langle r_2^n\rangle_{v_2}$, were already introduced. The surface defined by Eq. (6) is further denoted by $\langle V\rangle^{\mathrm{TE3}}_{v_1v_2}$. We performed the tests of $\langle V\rangle^{\mathrm{TE3}}_{v_1v_2}$ only for $v_1=0$ and $v_2=0$, with the reference values r_{ic}, $i=1,2$, chosen to be equal to the corresponding values of $\langle r_i\rangle_{v_i}$. Thus, the terms containing $(\langle r_2\rangle_{v_2}-r_{2c})$ and $(\langle r_1\rangle_{v_1}-r_{1c})$, i.e., related to $f^c_{21}(\boldsymbol{X})$ and $f^c_{12}(\boldsymbol{X})$, vanish. To calculate the remaining two third-order terms, comprising $f^c_{30}(\boldsymbol{X})$ and $f^c_{03}(\boldsymbol{X})$, we need to calculate these derivatives. To do this, we employed the following numerical formulas

$$f^c_{30}(\boldsymbol{X})\approx\frac{V(\boldsymbol{X},r_{1c}+2h_1,r_{2c})-2V(\boldsymbol{X},r_{1c}+h_1,r_{2c})+2V(\boldsymbol{X},r_{1c}-h_1,r_{2c})-V(\boldsymbol{X},r_{1c}-2h_1,r_{2c})}{2h_1^3},$$
$$f^c_{03}(\boldsymbol{X})\approx\frac{V(\boldsymbol{X},r_{1c},r_{2c}+2h_2)-2V(\boldsymbol{X},r_{1c},r_{2c}+h_2)+2V(\boldsymbol{X},r_{1c},r_{2c}-h_2)-V(\boldsymbol{X},r_{1c},r_{2c}-2h_2)}{2h_2^3},$$

where $h_1=h_2=0.025$ bohr. The values of $\langle V\rangle^{\mathrm{TE3}}_{00}$, defined by Eq. (6), were computed on-the-fly with the values of $\langle r_1^n\rangle_{v_1}$ and $\langle r_2^n\rangle_{v_2}$, $n=1,2$, listed in the main text. We needed also the averaged values of third powers of r_1 and r_2. Their values are $\langle r_2^3\rangle_0=9.82563$ bohr^3 and $\langle r_1^3\rangle_0=3.16163$ bohr^3 (3.17572 bohr^3) for the *para* (*ortho*) case. As we explained

elsewhere, we need different values of $\langle r_1^n \rangle_0$ to construct optimal $\langle V \rangle_{00}^{\mathrm{TE3}}$ surfaces for the *para* and *ortho* cases.

The leading term in the expansion (4), $f_{00}^c(\boldsymbol{X})$, is the interaction energy calculated at the reference intramonomer separations r_{1c} and r_{2c} bohr [32, 33] and it was taken directly from the 6D PES. The f_{ij}^c derivatives were calculated numerically using the 6D PES and the formulas listed in Ref. [33]. Such on-the-fly calculations are more expensive than with an analytic 4D PES, but since BOUND interpolates the interaction energy and use the interpolating function in actual rovibrational calculations, this increase in the costs is not an issue.

There is one interesting finding concerning results from the $V(\langle r_1 \rangle_0, \langle r_2 \rangle_0)$ and $\langle V \rangle_{00}^{\mathrm{TE}}$ surfaces. One may think that these surfaces should give predictions closer to each other than one can observe in Fig. 2A. This is because with our choice of the values of $r_{1c} = \langle r_1 \rangle_0 = 1.44835$ bohr and $r_{2c} = \langle r_2 \rangle_0 = 2.13993$ bohr in the definition of $\langle V \rangle_{00}^{\mathrm{TE}}$, where r_{ic} are the points around which the Taylor expansion is made, the linear terms and one of the quadratic terms, the one containing the product $(\langle r_1 \rangle_{v_1} - r_{1c})(\langle r_2 \rangle_{v_2} - r_{2c})$, vanish (for the $v_1 = v_2 = 0$ case). Still, the remaining quadratic terms do introduce corrections to $V(\langle r_1 \rangle_0, \langle r_2 \rangle_0)$ that make $\langle V \rangle_{00}^{\mathrm{TE}}$ quite significantly different and more accurate. The improvement of the reduced-dimensionality surface achieved in this way seems to be systematic, except for a couple of cases where the errors of $V(\langle r_1 \rangle_0, \langle r_2 \rangle_0)$ were fortuitously very small, as one can observe in the upper panel of Fig. 2A (at this scale, Δ_{TE} and Δ_{6D} are identical).

We have also performed rovibrational calculations for *ortho*D_2–CO complex. To construct the $V(\langle r_1 \rangle_0, \langle r_2 \rangle_0)$, $\langle V \rangle_{00}^{\mathrm{TE}}$, and $\langle V \rangle_{00}^{\mathrm{TE3}}$ surfaces we used the following averaged values of the r_1 coordinates: $\langle r_1 \rangle_0 = 1.43437$ bohr, $\langle r_1^2 \rangle_0 = 2.07713$ bohr^2, and $\langle r_1^3 \rangle_0 = 3.03637$ bohr^3, and we set $r_{1c} = \langle r_1 \rangle_0$.

SV. SYMMETRY OF ROVIBRATIONAL STATES

The rovibrational wave functions can be labelled by the total angular momentum quantum number, J, and by p, which is $+1$ for even parity and -1 for odd parity. Instead of p, one can use the spectroscopic parity defined in the case of H_2–CO as $P = (-1)^{J+j_1+j_2+l}$ with $P = +1 \equiv e$ and $P = -1 \equiv f$, where j_1 and j_2 denote the quantum numbers of the angular momenta of H_2 and CO, respectively, and l denotes the quantum number of the

end-over-end rotation of the complex. Although j_1, j_2, and l are only approximate quantum numbers and a given wave function can contain terms with several different values of each of these numbers, the sum $j_1 + j_2 + l$ is always either even or odd. The two parities are related by $P = (-1)^J p$. Note that since for J even $P = p$, but for J odd $P = -p$, even p parity can be either e or f parity. For each (J, P), there are several rovibrational states numbered by $n_{J,P}$. Thus, the states are unambiguously denoted by $(J, P, n_{J,P})$.

SVI. OVERALL OF ACCURACY OF REDUCED DIMENSIONALITY AND FULL-DIMENSIONALITY APPROACHES

When discussing the RMSEs of energy levels for *para*H_2–CO and *ortho*H_2–CO from 4D and 6D nuclear motion calculations relative to experiment, presented in Fig. 1 and Table SII, we have found that the RMSEs of levels computed with the $\langle V\rangle_{00}^{\mathrm{TE}}$, $\langle V\rangle_{00}^{\mathrm{TE3}}$, and $\langle V\rangle_{00}$ surfaces are equal to 0.0125 cm^{-1}, 0.0122 cm^{-1}, and 0.0119 cm^{-1}, respectively. What is surprising, is the fact that the complete 6D treatment results in a slightly larger RMSE, 0.0127 cm^{-1}. We have invested significant effort in checking the accuracy of the 6D nuclear dynamics calculations, as described in the relevant section of Methods, and are certain that both the 4D and 6D energy levels used in the current comparisons are accurate to 0.0001 cm^{-1}. This is an almost one order of magnitude smaller value than the difference between energy levels from $\langle V\rangle_{00}$ and V surfaces amounting to 0.0008 cm^{-1}. Thus, the discrepancies are meaningful. However, the other factor to take into account are uncertainties of the experimental line positions, which were estimated to be smaller than 0.0005 cm^{-1} [34], which can generate a maximum error of 0.001 cm^{-1} for an energy level. This value is similar to the difference between RMSEs for the levels from $\langle V\rangle_{00}$ and V, and this is the most likely explanation for the more approximate method giving results closer to experiment (in particular if the experimental uncertainties are underestimated). When comparing with experiment, it is also possible that experimental data are systematically offset. However, this cannot be the case here since we compare with energy levels, which are differences of measured transition energies.

SVII. SUPPLEMENTARY TABLES AND FIGURES

TABLE SI. The positions ($R_{\rm min}$) and values ($E_{\rm min}$) of the global ($(\theta_1, \theta_2, \phi) = (0^\circ, 180^\circ, 0^\circ)$) and local ($(\theta_1, \theta_2, \phi) = (0^\circ, 0^\circ, 0^\circ)$) minima for H_2–CO on the $V(r_{1e}, r_{2e})$, $V(\langle r_1 \rangle_0, \langle r_2 \rangle_0)$, $\langle V \rangle_{00}^{\rm TE}$, $\langle V \rangle_{00}^{\rm TE3}$, and $\langle V \rangle_{00}$ surfaces. The corresponding saddle points, with their positions, $(R, \theta_1, \theta_2, \phi)$, and values, $E_{\rm int}$, are also listed. The distances are given in bohr and energies in cm^{-1}.

4D surface	global minimum		local minimum		saddle point 1		saddle point 2	
	$R_{\rm min}$	$E_{\rm min}$	$R'_{\rm min}$	$E'_{\rm min}$	$(R, \theta_1, \theta_2, \phi)$	$E_{\rm int}$	$(R, \theta_1, \theta_2, \phi)$	$E_{\rm int}$
$V(r_{1e}, r_{2e})$	7.8955	-90.9390	7.1389	-71.9324	$(6.5579, 124.97^\circ, 71.09^\circ, 0^\circ)$	-66.7655	$(6.6413, 90^\circ, 94.63^\circ, 90^\circ)$	-54.1109
$V(\langle r_1 \rangle_0, \langle r_2 \rangle_0)$	7.9017	-94.2465	7.1552	-74.3878	$(6.5625, 124.59^\circ, 71.99^\circ, 0^\circ)$	-68.6867	$(6.6535, 90^\circ, 95.13^\circ, 90^\circ)$	-55.2923
$\langle V \rangle_{00}^{\rm TE}$	7.9082	-94.0310	7.1681	-73.8147	$(6.5694, 124.66^\circ, 71.79^\circ, 0^\circ)$	-68.3335	$(6.6562, 90^\circ, 95.15^\circ, 90^\circ)$	-55.0522
$\langle V \rangle_{00}^{\rm TE3}$	7.9082	-94.0128	7.1682	-73.7974	$(6.5695, 124.67^\circ, 71.77^\circ, 0^\circ)$	-68.3265	$(6.6562, 90^\circ, 95.14^\circ, 90^\circ)$	-55.0508
$\langle V \rangle_{00}$	7.9083	-94.0001	7.1682	-73.7875	$(6.5697, 124.69^\circ, 71.75^\circ, 0^\circ)$	-68.3298	$(6.6561, 90^\circ, 95.15^\circ, 90^\circ)$	-55.0576

TABLE SII. RMSEs and maximum deviations of calculated energy levels (relative to the lowest energy level of each case) from their experimental counterparts for *para*H_2–CO, *ortho*H_2–CO, and *ortho*D_2–CO (with $v_2 = 0$) [27, 34, 35]. The theoretical values were obtained in the 4D calculations with the $V(r_{1e}, r_{2e})$, $V(\langle r_1\rangle_0, \langle r_2\rangle_0)$, $\langle V\rangle_{00}^{\mathrm{TE}}$, $\langle V\rangle_{00}^{\mathrm{TE3}}$, and $\langle V\rangle_{00}$ surfaces, and in the 6D calculations with V. Only bound states are included in the comparison, except the $V(r_{1e}, r_{2e})$ case, where a few high-lying states became resonances, although they are bound in the other calculations. In the *ortho*H_2–CO case, the experimental energy levels were found for 90 bound states out of total 101 obtained from theory [27]. Similarly, 77 experimental energy levels were found in Ref. [35] out of 88 possible obtained from calculations in the present work. Energies are reported as wavenumbers in cm^{-1}.

	# states	RMSE	Max error
	4D calculations		
$V(r_{1e}, r_{2e})$			
paraH_2–CO	39[a]	0.0239	-0.1022
orthoH_2–CO	90[b]	0.1162	-0.3983
orthoD_2–CO	77[c]	0.0281	-0.1066
$V(\langle r_1\rangle_0, \langle r_2\rangle_0)$			
paraH_2–CO	39	0.0234	0.0472
orthoH_2–CO	90	0.0234	0.0640
orthoD_2–CO	77	0.0221	0.0423
$\langle V\rangle_{00}^{\mathrm{TE}}$			
paraH_2–CO	39	0.0049	-0.0143
orthoH_2–CO	90	0.0125	-0.0545
orthoD_2–CO	77	0.0053	-0.0129
$\langle V\rangle_{00}^{\mathrm{TE3}}$			
paraH_2–CO	39	0.0049	-0.0143
orthoH_2–CO	90	0.0122	-0.0537
orthoD_2–CO	77	0.0054	-0.0131
$\langle V\rangle_{00}$			
paraH_2–CO	39	0.0050	-0.0142
orthoH_2–CO	90	0.0119	-0.0530
orthoD_2–CO	77	0.0054	-0.0127
	6D calculations		
V			
paraH_2–CO	39	0.0052	-0.0143
orthoH_2–CO	90	0.0127	-0.0556

[a] Three states are quasibound.

[b] Three states are quasibound.

[c] Two states are quasibound.

TABLE SIII. The rovibrational energy levels for *para*H_2–CO obtained from the rigid-rotor calculations, $E^0_{r_e}$, $E^0_{\langle r\rangle}$, E^0_{TE}, E^0_{TE3}, and E^0_{av}, using five reduced-dimensionality surfaces: $V(r_{1e}, r_{2e})$, $V(\langle r_1\rangle_0, \langle r_2\rangle_0)$, $\langle V\rangle^{\mathrm{TE}}_{00}$, $\langle V\rangle^{\mathrm{TE3}}_{00}$, and $\langle V\rangle_{00}$, respectively, and from the full-dimensional calculations, E^0_{6D}, using the V surface. The values of these energies are compared with the experimental ones [34], E^0_{expt}, using the difference $\Delta_{\mathrm{method}} = E^0_{\mathrm{method}} - E^0_{\mathrm{expt}}$. The calculated energy levels are given relative to the ground level, $(J, P, n_{J,P}) = (0, e, 1)$, for each case, that is 3242.1956 cm^{-1} for E^0_{6D} and -18.8332 cm^{-1}, -19.5328 cm^{-1}, -19.4269 cm^{-1}, -19.4249 cm^{-1}, and -19.4262 cm^{-1} for $E^0_{r_e}$, $E^0_{\langle r\rangle}$, E^0_{TE}, E^0_{TE3}, and E^0_{av}, respectively, where the zero of energy is defined by H_2 and CO at infinite separation and in their ground states: $v_1 = v_2 = j_1 = j_2 = 0$. Only the bound states for $v_2 = 0$ are presented in this comparison, except for the three states marked with asterisks, which, in the case of the $V(r_{1e}, r_{2e})$ surface, shallower than the other surfaces, become the resonances. $n_{J,P}$ numbers consecutive energy levels. The values of Δ_{6D}, Δ_{r_e}, $\Delta_{\langle r\rangle}$, Δ_{TE}, Δ_{TE3}, and Δ_{av} are plotted in Fig. 2A. Energies are reported as wavenumbers in cm^{-1}.

J	P	$n_{J,P}$	E^0_{expt}	E^0_{6D}	Δ_{6D}	$E^0_{r_e}$	Δ_{r_e}	$E^0_{\langle r\rangle}$	$\Delta_{\langle r\rangle}$	E^0_{TE}	Δ_{TE}	E^0_{TE3}	Δ_{TE3}	E^0_{av}	Δ_{av}
0	e	1	0.0000	0.0000	0.0000	0.0000	0.0000	0.0000	0.0000	0.0000	0.0000	0.0000	0.0000	0.0000	0.0000
0	e	2	7.0794	7.0832	0.0038	7.0723	-0.0071	7.1131	0.0337	7.0832	0.0038	7.0830	0.0036	7.0842	0.0048
0	e	3	15.1685	15.1673	-0.0012	15.1057	-0.0628	15.1951	0.0266	15.1680	-0.0005	15.1677	-0.0008	15.1685	0.0000
1	f	1	4.0901	4.0829	-0.0072	4.1044	0.0143	4.0834	-0.0067	4.0833	-0.0068	4.0834	-0.0067	4.0831	-0.0070
1	f	2	15.6649	15.6655	0.0006	15.6711	0.0062	15.6929	0.0280	15.6657	0.0008	15.6656	0.0007	15.6663	0.0014
1	e	1	1.0538	1.0548	0.0010	1.0560	0.0022	1.0565	0.0027	1.0547	0.0009	1.0547	0.0009	1.0547	0.0009
1	e	2	3.6179	3.6116	-0.0063	3.6304	0.0125	3.6137	-0.0042	3.6120	-0.0059	3.6120	-0.0059	3.6119	-0.0060
1	e	3	8.4852	8.4890	0.0038	8.4811	-0.0041	8.5188	0.0336	8.4888	0.0036	8.4886	0.0034	8.4897	0.0045
1	e	4	13.6603	13.6602	-0.0001	13.6440	-0.0163	13.6847	0.0244	13.6609	0.0006	13.6607	0.0004	13.6616	0.0013
1	e	5	17.9132	17.9127	-0.0005	17.8110	-0.1022	17.9505	0.0373	17.9121	-0.0011	17.9117	-0.0015	17.9124	-0.0008
2	f	1	6.2657	6.2594	-0.0063	6.2844	0.0187	6.2641	-0.0016	6.2596	-0.0061	6.2596	-0.0061	6.2593	-0.0064
2	f	2	11.6901	11.6815	-0.0086	11.7090	0.0189	11.6827	-0.0074	11.6826	-0.0075	11.6827	-0.0074	11.6823	-0.0078
2	f	3	18.3415	18.3423	0.0008	18.3483	0.0068	18.3718	0.0303	18.3423	0.0008	18.3421	0.0006	18.3427	0.0012
2	e	1	3.1478	3.1496	0.0018	3.1524	0.0046	3.1546	0.0068	3.1493	0.0015	3.1492	0.0014	3.1493	0.0015
2	e	2	5.0143	5.0081	-0.0062	5.0282	0.0139	5.0160	0.0017	5.0084	-0.0059	5.0084	-0.0059	5.0084	-0.0059
2	e	3	11.0968	11.1008	0.0040	11.1024	0.0056	11.1289	0.0321	11.1005	0.0037	11.1003	0.0035	11.1011	0.0043
2	e	4	11.3665	11.3584	-0.0081	11.3719	0.0054	11.3664	-0.0001	11.3595	-0.0070	11.3595	-0.0070	11.3595	-0.0070
2	e	5	14.8066	14.8047	-0.0019	14.7956	-0.0110	14.8259	0.0193	14.8054	-0.0012	14.8053	-0.0013	14.8059	-0.0007
3	f	1	9.4899	9.4848	-0.0051	9.5140	0.0241	9.4961	0.0062	9.4847	-0.0052	9.4846	-0.0053	9.4844	-0.0055
3	f	2	14.0790	14.0721	-0.0069	14.1007	0.0217	14.0795	0.0005	14.0729	-0.0061	14.0729	-0.0061	14.0727	-0.0063
3	f	3	22.1106	22.1118	0.0012	22.1101	-0.0005	22.1463	0.0357	22.1114	0.0008	22.1112	0.0006	22.1118	0.0012
3	f	4	23.1637	23.1548	-0.0089	23.1761*	0.0124	23.1579	-0.0058	23.1573	-0.0064	23.1573	-0.0064	23.1570	-0.0067
3	e	1	6.2482	6.2522	0.0040	6.2548	0.0066	6.2618	0.0136	6.2516	0.0034	6.2515	0.0033	6.2516	0.0034
3	e	2	7.2927	7.2863	-0.0064	7.3111	0.0184	7.3021	0.0094	7.2865	-0.0062	7.2864	-0.0063	7.2865	-0.0062
3	e	3	12.9333	12.9300	-0.0033	12.9435	0.0102	12.9429	0.0096	12.9309	-0.0024	12.9309	-0.0024	12.9311	-0.0022
3	e	4	14.8213	14.8239	0.0026	14.8148	-0.0065	14.8608	0.0395	14.8232	0.0019	14.8230	0.0017	14.8238	0.0025
3	e	5	17.5013	17.4989	-0.0024	17.4990	-0.0023	17.5198	0.0185	17.4995	-0.0018	17.4993	-0.0020	17.4996	-0.0017
4	f	1	13.7090	13.7047	-0.0043	13.7365	0.0275	13.7253	0.0163	13.7042	-0.0048	13.7041	-0.0049	13.7039	-0.0051
4	f	2	17.4032	17.3986	-0.0046	17.4276	0.0244	17.4135	0.0103	17.3991	-0.0041	17.3991	-0.0041	17.3990	-0.0042
4	e	1	10.2717	10.2848	0.0131	10.2697	-0.0020	10.2936	0.0219	10.2837	0.0120	10.2836	0.0119	10.2837	0.0120
4	e	2	10.5174	10.5031	-0.0143	10.5485	0.0311	10.5356	0.0182	10.5031	-0.0143	10.5030	-0.0144	10.5032	-0.0142
4	e	3	15.2555	15.2543	-0.0012	15.2607	0.0052	15.2747	0.0192	15.2549	-0.0006	15.2548	-0.0007	15.2552	-0.0003
4	e	4	19.4112	19.4139	0.0027	19.3912*	-0.0200	19.4584	0.0472	19.4127	0.0015	19.4123	0.0011	19.4131	0.0019
5	f	1	18.8312	18.8277	-0.0035	18.8559	0.0247	18.8607	0.0295	18.8267	-0.0045	18.8264	-0.0048	18.8264	-0.0048
5	f	2	21.6497	21.6474	-0.0023	21.6728	0.0231	21.6709	0.0212	21.6474	-0.0023	21.6473	-0.0024	21.6473	-0.0024
5	e	1	14.5240	14.5238	-0.0002	14.5354	0.0114	14.5526	0.0286	14.5232	-0.0008	14.5230	-0.0010	14.5233	-0.0007
5	e	2	15.2730	15.2729	-0.0001	15.2870	0.0140	15.3058	0.0328	15.2717	-0.0013	15.2714	-0.0016	15.2716	-0.0014
5	e	3	18.4259	18.4275	0.0016	18.4256	-0.0003	18.4554	0.0295	18.4278	0.0019	18.4276	0.0017	18.4282	0.0023
6	e	1	19.4006	19.3973	-0.0033	19.4065*	0.0059	19.4419	0.0413	19.3963	-0.0043	19.3960	-0.0046	19.3965	-0.0041

TABLE SIV. The rovibrational energy levels for *para*H_2–CO obtained from the rigid-rotor calculations, $E^0_{r_e}$, $E^0_{\langle r\rangle}$, E^0_{TE}, E^0_{TE3}, and E^0_{av}, using five reduced-dimensionality surfaces: $V(r_{1e}, r_{2e})$, $V(\langle r_1\rangle_0, \langle r_2\rangle_0)$, $\langle V\rangle^{\mathrm{TE}}_{00}$, $\langle V\rangle^{\mathrm{TE3}}_{00}$, and $\langle V\rangle_{00}$, respectively, and from the full-dimensional calculations, E^0_{6D}, using the V surface. All the energies are listed also in Table SIII, but here we compare the energies obtained from the reduced-dimensionality calculations with those from the full-dimensional ones. The differences are denoted as $\Delta^{\mathrm{6D}}_{\mathrm{method}} = E^0_{\mathrm{method}} - E^0_{\mathrm{6D}}$. All other information is specified in Table SIII. The values of the RMSE and the maximum discrepancies of the reduced-dimensionality energies from the full-dimensionality ones are also given. Energies are reported as wavenumbers in cm^{-1}.

J	P	$n_{J,P}$	E^0_{6D}	$E^0_{r_e}$	$\Delta^{\mathrm{6D}}_{r_e}$	$E^0_{\langle r\rangle}$	$\Delta^{\mathrm{6D}}_{\langle r\rangle}$	E^0_{TE}	$\Delta^{\mathrm{6D}}_{\mathrm{TE}}$	E^0_{TE3}	$\Delta^{\mathrm{6D}}_{\mathrm{TE3}}$	E^0_{av}	$\Delta^{\mathrm{6D}}_{\mathrm{av}}$
0	e	1	0.0000	0.0000	0.0000	0.0000	0.0000	0.0000	0.0000	0.0000	0.0000	0.0000	0.0000
0	e	2	7.0832	7.0723	-0.0109	7.1131	0.0299	7.0832	0.0000	7.0830	-0.0002	7.0842	0.0010
0	e	3	15.1673	15.1057	-0.0616	15.1951	0.0278	15.1680	0.0007	15.1677	0.0004	15.1685	0.0012
1	f	1	4.0829	4.1044	0.0215	4.0834	0.0005	4.0833	0.0004	4.0834	0.0005	4.0831	0.0002
1	f	2	15.6655	15.6711	0.0056	15.6929	0.0274	15.6657	0.0002	15.6656	0.0001	15.6663	0.0008
1	e	1	1.0548	1.0560	0.0012	1.0565	0.0017	1.0547	-0.0001	1.0547	-0.0001	1.0547	-0.0001
1	e	2	3.6116	3.6304	0.0188	3.6137	0.0021	3.6120	0.0004	3.6120	0.0004	3.6119	0.0003
1	e	3	8.4890	8.4811	-0.0079	8.5188	0.0298	8.4888	-0.0002	8.4886	-0.0004	8.4897	0.0007
1	e	4	13.6602	13.6440	-0.0162	13.6847	0.0245	13.6609	0.0007	13.6607	0.0005	13.6616	0.0014
1	e	5	17.9127	17.8110	-0.1017	17.9505	0.0378	17.9121	-0.0006	17.9117	-0.0010	17.9124	-0.0003
2	f	1	6.2594	6.2844	0.0250	6.2641	0.0047	6.2596	0.0002	6.2596	0.0002	6.2593	-0.0001
2	f	2	11.6815	11.7090	0.0275	11.6827	0.0012	11.6826	0.0011	11.6827	0.0012	11.6823	0.0008
2	f	3	18.3423	18.3483	0.0060	18.3718	0.0295	18.3423	0.0000	18.3421	-0.0002	18.3427	0.0004
2	e	1	3.1496	3.1524	0.0028	3.1546	0.0050	3.1493	-0.0003	3.1492	-0.0004	3.1493	-0.0003
2	e	2	5.0081	5.0282	0.0201	5.0160	0.0079	5.0084	0.0003	5.0084	0.0003	5.0084	0.0003
2	e	3	11.1008	11.1024	0.0016	11.1289	0.0281	11.1005	-0.0003	11.1003	-0.0005	11.1011	0.0003
2	e	4	11.3584	11.3719	0.0135	11.3664	0.0080	11.3595	0.0011	11.3595	0.0011	11.3595	0.0011
2	e	5	14.8047	14.7956	-0.0091	14.8259	0.0212	14.8054	0.0007	14.8053	0.0006	14.8059	0.0012
3	f	1	9.4848	9.5140	0.0292	9.4961	0.0113	9.4847	-0.0001	9.4846	-0.0002	9.4844	-0.0004
3	f	2	14.0721	14.1007	0.0286	14.0795	0.0074	14.0729	0.0008	14.0729	0.0008	14.0727	0.0006
3	f	3	22.1118	22.1101	-0.0017	22.1463	0.0345	22.1114	-0.0004	22.1112	-0.0006	22.1118	0.0000
3	f	4	23.1548	23.1761*	0.0213	23.1579	0.0031	23.1573	0.0025	23.1573	0.0025	23.1570	0.0022
3	e	1	6.2522	6.2548	0.0026	6.2618	0.0096	6.2516	-0.0006	6.2515	-0.0007	6.2516	-0.0006
3	e	2	7.2863	7.3111	0.0248	7.3021	0.0158	7.2865	0.0002	7.2864	0.0001	7.2865	0.0002
3	e	3	12.9300	12.9435	0.0135	12.9429	0.0129	12.9309	0.0009	12.9309	0.0009	12.9311	0.0011
3	e	4	14.8239	14.8148	-0.0091	14.8608	0.0369	14.8232	-0.0007	14.8230	-0.0009	14.8238	-0.0001
3	e	5	17.4989	17.4990	0.0001	17.5198	0.0209	17.4995	0.0006	17.4993	0.0004	17.4996	0.0007
4	f	1	13.7047	13.7365	0.0318	13.7253	0.0206	13.7042	-0.0005	13.7041	-0.0006	13.7039	-0.0008
4	f	2	17.3986	17.4276	0.0290	17.4135	0.0149	17.3991	0.0005	17.3991	0.0005	17.3990	0.0004
4	e	1	10.2848	10.2697	-0.0151	10.2936	0.0088	10.2837	-0.0011	10.2836	-0.0012	10.2837	-0.0011
4	e	2	10.5031	10.5485	0.0454	10.5356	0.0325	10.5031	0.0000	10.5030	-0.0001	10.5032	0.0001
4	e	3	15.2543	15.2607	0.0064	15.2747	0.0204	15.2549	0.0006	15.2548	0.0005	15.2552	0.0009
4	e	4	19.4139	19.3912*	-0.0227	19.4584	0.0445	19.4127	-0.0012	19.4123	-0.0016	19.4131	-0.0008
5	f	1	18.8277	18.8559	0.0282	18.8607	0.0330	18.8267	-0.0010	18.8264	-0.0013	18.8264	-0.0013
5	f	2	21.6474	21.6728	0.0254	21.6709	0.0235	21.6474	0.0000	21.6473	-0.0001	21.6473	-0.0001
5	e	1	14.5238	14.5354	0.0116	14.5526	0.0288	14.5232	-0.0006	14.5230	-0.0008	14.5233	-0.0005
5	e	2	15.2729	15.2870	0.0141	15.3058	0.0329	15.2717	-0.0012	15.2714	-0.0015	15.2716	-0.0013
5	e	3	18.4275	18.4256	-0.0019	18.4554	0.0279	18.4278	0.0003	18.4276	0.0001	18.4282	0.0007
6	e	1	19.3973	19.4065*	0.0092	19.4419	0.0446	19.3963	-0.0010	19.3960	-0.0013	19.3965	-0.0008
RMSE					0.0264		0.0236		0.0007		0.0008		0.0008
Max. error					-0.1017		0.0446		0.0025		0.0025		0.0022

TABLE SV. The even-parity rovibrational energy levels for *ortho*H_2–CO obtained from the rigid-rotor calculations, $E^0_{r_e}$, $E^0_{\langle r\rangle}$, E^0_{TE}, E^0_{TE3}, and E^0_{av}, using five reduced-dimensionality surfaces: $V(r_{1e}, r_{2e})$, $V(\langle r_1\rangle_0, \langle r_2\rangle_0)$, $\langle V\rangle^{\mathrm{TE}}_{00}$, $\langle V\rangle^{\mathrm{TE3}}_{00}$, and $\langle V\rangle_{00}$, respectively, and from the full-dimensional calculations, E^0_{6D}, using the V surface. The values of these energies are compared with the experimental ones [27], E^0_{expt}, using the difference $\Delta_{\mathrm{method}} = E^0_{\mathrm{method}} - E^0_{\mathrm{expt}}$. For each case, the calculated energy levels are given relative to the $(J, P, n_{J,P}) = (1, f, 1)$ level, that is 3360.2251 cm^{-1} for E^0_{6D} and 99.5500 cm^{-1}, 98.6839 cm^{-1}, 98.7931 cm^{-1}, 98.7952 cm^{-1}, and 98.7933 cm^{-1} for $E^0_{r_e}$, $E^0_{\langle r\rangle}$, E^0_{TE}, E^0_{TE3}, and E^0_{av}, respectively, where the zero of energy is defined by H_2 and CO at infinite separation and in their ground states: $v_1 = v_2 = j_1 = j_2 = 0$. The reference 4D energies are positive because the dissociation limit for $j_1 = 1$ is at 118.6766 cm^{-1}. Only the bound states for $v_2 = 0$ are presented in this comparison, except for one state for the $V(r_{1e}, r_{2e})$ surface, marked with asterisk, which becomes a resonance. $n_{J,P}$ numbers consecutive energy levels. The values of Δ_{6D}, Δ_{r_e}, $\Delta_{\langle r\rangle}$, Δ_{TE}, Δ_{TE3}, and Δ_{av} are plotted in Fig. 2B. Energies are reported as wavenumbers in cm^{-1}.

J	P	$n_{J,P}$	E^0_{expt}	E^0_{6D}	Δ_{6D}	$E^0_{r_e}$	Δ_{r_e}	$E^0_{\langle r\rangle}$	$\Delta_{\langle r\rangle}$	E^0_{TE}	Δ_{TE}	E^0_{TE3}	Δ_{TE3}	E^0_{av}	Δ_{av}
0	e	1	-0.4146	-0.4350	-0.0204	-0.3247	0.0899	-0.4266	-0.0120	-0.4353	-0.0207	-0.4338	-0.0192	-0.4312	-0.0166
0	e	2	0.3488	0.3373	-0.0115	0.5358	0.1870	0.3265	-0.0223	0.3378	-0.0110	0.3390	-0.0098	0.3398	-0.0090
0	e	3		3.5005		3.5573		3.5076		3.5009		3.5020		3.5042	
0	e	4		12.2427		12.2543		12.2520		12.2434		12.2443		12.2463	
0	e	5		18.0695		17.8491		18.1029		18.0687		18.0681		18.0692	
1	f	1	0.0000	0.0000	0.0000	0.0000	0.0000	0.0000	0.0000	0.0000	0.0000	0.0000	0.0000	0.0000	0.0000
1	f	2	3.5320	3.5249	-0.0071	3.4939	-0.0381	3.5314	-0.0006	3.5245	-0.0075	3.5250	-0.0070	3.5261	-0.0059
1	f	3	6.7695	6.7646	-0.0049	6.6648	-0.1047	6.7727	0.0032	6.7640	-0.0055	6.7636	-0.0059	6.7637	-0.0058
1	f	4	10.7296	10.7340	0.0044	10.6836	-0.0460	10.7308	0.0012	10.7344	0.0048	10.7342	0.0046	10.7341	0.0045
1	f	5	12.4507	12.4457	-0.0050	12.2895	-0.1612	12.4592	0.0085	12.4459	-0.0048	12.4463	-0.0044	12.4481	-0.0026
1	f	6	12.7442	12.7540	0.0098	12.5758	-0.1684	12.7924	0.0482	12.7528	0.0086	12.7518	0.0076	12.7528	0.0086
1	f	7	18.6178	18.6217	0.0039	18.2195	-0.3983	18.6818	0.0640	18.6189	0.0011	18.6177	-0.0001	18.6192	0.0014
1	f	8		19.8199		19.5409*		19.8788		19.8170		19.8155		19.8166	
2	e	1	0.4335	0.4337	0.0002	0.4173	-0.0162	0.4354	0.0019	0.4333	-0.0002	0.4337	0.0002	0.4347	0.0012
2	e	2	1.9017	1.8760	-0.0257	2.0095	0.1078	1.8862	-0.0155	1.8764	-0.0253	1.8774	-0.0243	1.8787	-0.0230
2	e	3	3.3543	3.3301	-0.0242	3.4872	0.1329	3.3411	-0.0132	3.3304	-0.0239	3.3315	-0.0228	3.3329	-0.0214
2	e	4	3.8509	3.8582	0.0073	3.9109	0.0600	3.8480	-0.0029	3.8583	0.0074	3.8586	0.0077	3.8588	0.0079
2	e	5	5.8457	5.8403	-0.0054	5.8945	0.0488	5.8449	-0.0008	5.8404	-0.0053	5.8412	-0.0045	5.8426	-0.0031
2	e	6	6.9211	6.9136	-0.0075	6.9215	0.0004	6.9313	0.0102	6.9133	-0.0078	6.9139	-0.0072	6.9158	-0.0053
2	e	7	11.3467	11.3420	-0.0047	11.3200	-0.0267	11.3445	-0.0022	11.3421	-0.0046	11.3427	-0.0040	11.3437	-0.0030
2	e	8	12.0851	12.0814	-0.0037	12.0686	-0.0165	12.0892	0.0041	12.0822	-0.0029	12.0826	-0.0025	12.0838	-0.0013
2	e	9	13.5362	13.5445	0.0083	13.4224	-0.1138	13.5584	0.0222	13.5440	0.0078	13.5439	0.0077	13.5450	0.0088
2	e	10	16.2895	16.2950	0.0055	16.0723	-0.2172	16.3377	0.0482	16.2935	0.0040	16.2927	0.0032	16.2939	0.0044
2	e	11		17.1042		17.0139		17.1304		17.1038		17.1040		17.1058	
2	e	12	18.6931	18.7015	0.0084	18.4423	-0.2508	18.7382	0.0451	18.7006	0.0075	18.6997	0.0066	18.7006	0.0075
3	f	1	5.2771	5.2768	-0.0003	5.2875	0.0104	5.2851	0.0080	5.2764	-0.0007	5.2765	-0.0006	5.2766	-0.0005
3	f	2	6.0931	6.0878	-0.0053	6.0519	-0.0412	6.0943	0.0012	6.0878	-0.0053	6.0877	-0.0054	6.0877	-0.0054
3	f	3	6.9004	6.8970	-0.0034	6.8531	-0.0473	6.9093	0.0089	6.8964	-0.0040	6.8965	-0.0039	6.8972	-0.0032
3	f	4	11.0419	11.0571	0.0152	11.0300	-0.0119	11.0573	0.0154	11.0580	0.0161	11.0578	0.0159	11.0575	0.0156
3	f	5		12.1527		12.1244		12.1623		12.1529		12.1532		12.1540	
3	f	6	13.7462	13.7450	-0.0012	13.6947	-0.0515	13.7631	0.0169	13.7440	-0.0022	13.7439	-0.0023	13.7444	-0.0018
3	f	7	16.6636	16.6686	0.0050	16.6037	-0.0599	16.6863	0.0227	16.6686	0.0050	16.6683	0.0047	16.6687	0.0051
3	f	8	17.6031	17.6098	0.0067	17.5071	-0.0960	17.6336	0.0305	17.6094	0.0063	17.6095	0.0064	17.6110	0.0079
3	f	9	18.1914	18.2096	0.0182	17.9848	-0.2066	18.2472	0.0558	18.2080	0.0166	18.2072	0.0158	18.2083	0.0169
4	e	1	5.8976	5.8889	-0.0087	5.8642	-0.0334	5.9114	0.0138	5.8883	-0.0093	5.8887	-0.0089	5.8899	-0.0077
4	e	2	6.0155	6.0044	-0.0111	6.0689	0.0534	6.0153	-0.0002	6.0040	-0.0115	6.0047	-0.0108	6.0061	-0.0094
4	e	3	9.0323	9.0053	-0.0270	9.2094	0.1771	9.0222	-0.0101	9.0058	-0.0265	9.0071	-0.0252	9.0090	-0.0233
4	e	4	11.1984	11.2039	0.0055	11.2900	0.0916	11.2059	0.0075	11.2037	0.0053	11.2042	0.0058	11.2048	0.0064
4	e	5	12.3467	12.3384	-0.0083	12.4292	0.0825	12.3537	0.0070	12.3383	-0.0084	12.3389	-0.0078	12.3398	-0.0069
4	e	6	13.9806	13.9782	-0.0024	13.9541	-0.0265	14.0111	0.0305	13.9773	-0.0033	13.9776	-0.0030	13.9792	-0.0014
4	e	7	14.6812	14.6795	-0.0017	14.6492	-0.0320	14.6869	0.0057	14.6799	-0.0013	14.6797	-0.0015	14.6796	-0.0016
4	e	8	16.4405	16.4381	-0.0024	16.4665	0.0260	16.4517	0.0112	16.4381	-0.0024	16.4388	-0.0017	16.4403	-0.0002
4	e	9	17.2422	17.2408	-0.0014	17.2013	-0.0409	17.2557	0.0135	17.2410	-0.0012	17.2411	-0.0011	17.2419	-0.0003
4	e	10	19.6146	19.6306	0.0160	19.6640	0.0494	19.6713	0.0567	19.6288	0.0142	19.6280	0.0134	19.6291	0.0145
5	f	1	13.9873	13.9675	-0.0198	13.9313	-0.0560	14.0082	0.0209	13.9664	-0.0209	13.9665	-0.0208	13.9674	-0.0199
5	f	2	14.1405	14.1413	0.0008	14.1522	0.0117	14.1621	0.0216	14.1407	0.0002	14.1406	0.0001	14.1409	0.0004
5	f	3	14.5351	14.5496	0.0145	14.4851	-0.0500	14.5616	0.0265	14.5486	0.0135	14.5484	0.0133	14.5486	0.0135
5	f	4	17.7509	17.7489	-0.0020	17.6721	-0.0788	17.7673	0.0164	17.7485	-0.0024	17.7484	-0.0025	17.7492	-0.0017
5	f	5	18.5479	18.5513	0.0034	18.4666	-0.0813	18.5723	0.0244	18.5512	0.0033	18.5508	0.0029	18.5510	0.0031
6	e	1	13.4622	13.4327	-0.0295	13.4918	0.0296	13.4793	0.0171	13.4322	-0.0300	13.4328	-0.0294	13.4346	-0.0276
6	e	2	15.0840	15.0927	0.0087	15.0325	-0.0515	15.1230	0.0390	15.0909	0.0069	15.0910	0.0070	15.0922	0.0082
6	e	3	16.1165	16.1048	-0.0117	16.2124	0.0959	16.1231	0.0066	16.1048	-0.0117	16.1058	-0.0107	16.1075	-0.0090

TABLE SVI. The odd-parity rovibrational energy levels for *ortho*H_2–CO obtained from the rigid-rotor calculations, $E^0_{r_e}$, $E^0_{\langle r\rangle}$, $E^0_{\rm TE}$, $E^0_{\rm TE3}$, and $E^0_{\rm av}$, using five reduced-dimensionality surfaces: $V(r_{1e}, r_{2e})$, $V(\langle r_1\rangle_0, \langle r_2\rangle_0)$, $\langle V\rangle^{\rm TE}_{00}$, $\langle V\rangle^{\rm TE3}_{00}$, and $\langle V\rangle_{00}$, respectively, and from the full-dimensional calculations, $E^0_{\rm 6D}$, using the V surface. The values of these energies are compared with the experimental ones [27], $E^0_{\rm expt}$, using the difference $\Delta_{\rm method} = E^0_{\rm method} - E^0_{\rm expt}$. The calculated energy levels are given relative to the lowest level of the even parity, $(J, P, n_{J,P}) = (1, e, 1)$, for each case, that is 3359.4071 cm^{-1} for $E^0_{\rm 6D}$ and 98.7283 cm^{-1}, 97.8650 cm^{-1}, 97.9748 cm^{-1}, 97.9773 cm^{-1}, and 97.9763 cm^{-1} for $E^0_{r_e}$, $E^0_{\langle r\rangle}$, $E^0_{\rm TE}$, $E^0_{\rm TE3}$, and $E^0_{\rm av}$, respectively, where the zero of energy is defined by H_2 and CO at infinite separation and in their ground states: $v_1 = v_2 = j_1 = j_2 = 0$. The reference 4D energies are positive because the dissociation limit for $j_1 = 1$ is at 118.6766 cm^{-1}. Only the bound states for $v_2 = 0$ are presented in this comparison, except for two states for the $V(r_{1e}, r_{2e})$ surface, marked with asterisks, which become resonances. $n_{J,P}$ numbers consecutive energy levels. The values of $\Delta_{\rm 6D}$, Δ_{r_e}, $\Delta_{\langle r\rangle}$, $\Delta_{\rm TE}$, $\Delta_{\rm TE3}$, and $\Delta_{\rm av}$ are plotted in Fig. S2. Energies are reported as wavenumbers in cm^{-1}.

J	P	$n_{J,P}$	$E^0_{\rm expt}$	$E^0_{\rm 6D}$	$\Delta_{\rm 6D}$	$E^0_{r_e}$	Δ_{r_e}	$E^0_{\langle r\rangle}$	$\Delta_{\langle r\rangle}$	$E^0_{\rm TE}$	$\Delta_{\rm TE}$	$E^0_{\rm TE3}$	$\Delta_{\rm TE3}$	$E^0_{\rm av}$	$\Delta_{\rm av}$
0	f	1	5.7009	5.7036	0.0027	5.5549	-0.1460	5.7083	0.0074	5.7033	0.0024	5.7022	0.0013	5.7009	0.0000
0	f	2	19.0004	18.9930	-0.0074	18.7392	-0.2612	19.0280	0.0276	18.9921	-0.0083	18.9906	-0.0098	18.9904	-0.0100
0	f	3		24.4935		23.7888		24.5912		24.4881		24.4857		24.4865	
1	e	1	0.0000	0.0000	0.0000	0.0000	0.0000	0.0000	0.0000	0.0000	0.0000	0.0000	0.0000	0.0000	0.0000
1	e	2	1.7322	1.6766	-0.0556	1.8975	0.1653	1.7088	-0.0234	1.6777	-0.0545	1.6785	-0.0537	1.6792	-0.0530
1	e	3	1.9991	2.0260	0.0269	2.1350	0.1359	1.9982	-0.0009	2.0258	0.0267	2.0266	0.0275	2.0271	0.0280
1	e	4	4.8568	4.8512	-0.0056	4.8921	0.0353	4.8568	0.0000	4.8514	-0.0054	4.8519	-0.0049	4.8527	-0.0041
1	e	5	5.5794	5.5737	-0.0057	5.6036	0.0242	5.5865	0.0071	5.5737	-0.0057	5.5742	-0.0052	5.5754	-0.0040
1	e	6	11.5687	11.5725	0.0038	11.5121	-0.0566	11.5739	0.0052	11.5734	0.0047	11.5727	0.0040	11.5717	0.0030
1	e	7	12.3718	12.3687	-0.0031	12.3498	-0.0220	12.3739	0.0021	12.3690	-0.0028	12.3696	-0.0022	12.3709	-0.0009
1	e	8	14.1039	14.1146	0.0107	13.8817	-0.2222	14.1549	0.0510	14.1135	0.0096	14.1122	0.0083	14.1125	0.0086
1	e	9		15.0844		15.0577		15.1019		15.0849		15.0850		15.0861	
1	e	10	18.4528	18.4510	-0.0018	18.1310	-0.3218	18.4959	0.0431	18.4499	-0.0029	18.4483	-0.0045	18.4486	-0.0042
1	e	11		20.3216		19.7214		20.4091		20.3171		20.3151		20.3161	
2	f	1	2.9497	2.9509	0.0012	2.9586	0.0089	2.9550	0.0053	2.9509	0.0012	2.9506	0.0009	2.9498	0.0001
2	f	2	4.2164	4.2139	-0.0025	4.1987	-0.0177	4.2154	-0.0010	4.2144	-0.0020	4.2139	-0.0025	4.2129	-0.0035
2	f	3	5.5170	5.5138	-0.0032	5.4753	-0.0417	5.5235	0.0065	5.5135	-0.0035	5.5134	-0.0036	5.5135	-0.0035
2	f	4	10.5879	10.5843	-0.0036	10.5140	-0.0739	10.6004	0.0125	10.5837	-0.0042	10.5831	-0.0048	10.5827	-0.0052
2	f	5	11.5902	11.5880	-0.0022	11.5705	-0.0197	11.5898	-0.0004	11.5885	-0.0017	11.5885	-0.0017	11.5885	-0.0017
2	f	6	14.2658	14.2711	0.0053	14.2137	-0.0521	14.2829	0.0171	14.2715	0.0057	14.2709	0.0051	14.2705	0.0047
2	f	7	15.3468	15.3448	-0.0020	15.2310	-0.1158	15.3636	0.0168	15.3450	-0.0018	15.3448	-0.0020	15.3456	-0.0012
2	f	8	15.7942	15.8112	0.0170	15.5999	-0.1943	15.8485	0.0543	15.8100	0.0158	15.8087	0.0145	15.8090	0.0148
2	f	9	19.4981	19.4950	-0.0031	19.1463	-0.3518	19.5415	0.0434	19.4936	-0.0045	19.4921	-0.0060	19.4926	-0.0055
3	e	1	3.4907	3.4958	0.0051	3.4734	-0.0173	3.5034	0.0127	3.4954	0.0047	3.4954	0.0047	3.4956	0.0049
3	e	2	4.3682	4.3480	-0.0202	4.4478	0.0796	4.3620	-0.0062	4.3485	-0.0197	4.3489	-0.0193	4.3495	-0.0187
3	e	3	6.8674	6.8389	-0.0285	7.0479	0.1805	6.8568	-0.0106	6.8395	-0.0279	6.8404	-0.0270	6.8412	-0.0262
3	e	4	7.9297	7.9389	0.0092	8.0058	0.0761	7.9343	0.0046	7.9390	0.0093	7.9390	0.0093	7.9384	0.0087
3	e	5	9.4459	9.4446	-0.0013	9.5266	0.0807	9.4524	0.0065	9.4449	-0.0010	9.4452	-0.0007	9.4456	-0.0003
3	e	6	10.8121	10.8110	-0.0011	10.8068	-0.0053	10.8366	0.0245	10.8106	-0.0015	10.8107	-0.0014	10.8117	-0.0004
3	e	7	11.9413	11.9389	-0.0024	11.9197	-0.0216	11.9403	-0.0010	11.9399	-0.0014	11.9394	-0.0019	11.9382	-0.0031
3	e	8	14.1776	14.1731	-0.0045	14.1717	-0.0059	14.1816	0.0040	14.1734	-0.0042	14.1736	-0.0040	14.1742	-0.0034
3	e	9	14.9359	14.9360	0.0001	14.9167	-0.0192	14.9469	0.0110	14.9368	0.0009	14.9367	0.0008	14.9369	0.0010
3	e	10	16.9599	16.9755	0.0156	16.7698	-0.1901	17.0039	0.0440	16.9747	0.0148	16.9737	0.0138	16.9739	0.0140
4	f	1	10.1616	10.1623	0.0007	10.1887	0.0271	10.1791	0.0175	10.1620	0.0004	10.1616	0.0000	10.1610	-0.0006
4	f	2	10.4768	10.4706	-0.0062	10.4247	-0.0521	10.4894	0.0126	10.4703	-0.0065	10.4698	-0.0070	10.4694	-0.0074
4	f	3	10.9566	10.9619	0.0053	10.9146	-0.0420	10.9763	0.0197	10.9613	0.0047	10.9608	0.0042	10.9606	0.0040
4	f	4	15.0416	15.0411	-0.0005	14.9713	-0.0703	15.0501	0.0085	15.0416	0.0000	15.0410	-0.0006	15.0404	-0.0012
4	f	5		15.4598		15.4370		15.4748		15.4602		15.4598		15.4596	
4	f	6	19.2917	19.2933	0.0016	19.2701	-0.0216	19.3207	0.0290	19.2924	0.0007	19.2920	0.0003	19.2918	0.0001
5	e	1	10.1430	10.1202	-0.0228	10.1889	0.0459	10.1547	0.0117	10.1202	-0.0228	10.1205	-0.0225	10.1214	-0.0216
5	e	2	10.8711	10.8803	0.0092	10.8378	-0.0333	10.8999	0.0288	10.8792	0.0081	10.8789	0.0078	10.8793	0.0082
5	e	3	13.1214	13.1073	-0.0141	13.2713	0.1499	13.1249	0.0035	13.1079	-0.0135	13.1086	-0.0128	13.1097	-0.0117
5	e	4	16.5019	16.5009	-0.0010	16.6501	0.1482	16.5133	0.0114	16.5012	-0.0007	16.5016	-0.0003	16.5020	0.0001
5	e	5	17.9921	17.9889	-0.0032	18.0526	0.0605	18.0161	0.0240	17.9884	-0.0037	17.9882	-0.0039	17.9880	-0.0041
5	e	6		19.6326		19.5909		19.6765		19.6315		19.6312		19.6319	
5	e	7	20.0810	20.0881	0.0071	20.0786*	-0.0024	20.1026	0.0216	20.0883	0.0073	20.0878	0.0068	20.0872	0.0062
6	f	1	19.7898	19.7704	-0.0194	19.7499	-0.0399	19.8261	0.0363	19.7692	-0.0206	19.7687	-0.0211	19.7690	-0.0208
6	f	2	20.3093	20.3159	0.0066	20.2641*	-0.0452	20.3475	0.0382	20.3150	0.0057	20.3143	0.0050	20.3139	0.0046
7	e	1	19.1340	19.1021	-0.0319	19.1617	0.0277	19.1662	0.0322	19.1016	-0.0324	19.1016	-0.0324	19.1028	-0.0312

TABLE SVII. The rovibrational energy levels for *ortho*H_2–CO obtained from the rigid-rotor calculations, $E^0_{r_e}$, $E^0_{\langle r\rangle}$, $E^0_{\rm TE}$, $E^0_{\rm TE3}$, and $E^0_{\rm av}$, using five reduced-dimensionality surfaces: $V(r_{1e}, r_{2e})$, $V(\langle r_1\rangle_0, \langle r_2\rangle_0)$, $\langle V\rangle^{\rm TE}_{00}$, $\langle V\rangle^{\rm TE3}_{00}$, and $\langle V\rangle_{00}$, respectively, and from the full-dimensional calculations, $E^0_{\rm 6D}$, using the V surface. The energies $E^0_{\rm 6D}$ and $E^0_{\rm method}$ are listed also in Tables SV and SVI, but here all of them are given relative to the lowest level $(J, P, n_{J,P}) = (1, e, 1)$, for each case. We compare the energies obtained from the reduced-dimensionality calculations with those from the full-dimensional ones by the following difference $\Delta^{\rm 6D}_{\rm method} = E^0_{\rm method} - E^0_{\rm 6D}$. All other information is specified in Table SVI. Energies are reported as wavenumbers in cm^{-1}.

J	P	$n_{J,P}$	$E^0_{\rm 6D}$	$E^0_{r_e}$	$\Delta^{\rm 6D}_{r_e}$	$E^0_{\langle r\rangle}$	$\Delta^{\rm 6D}_{\langle r\rangle}$	$E^0_{\rm TE}$	$\Delta^{\rm 6D}_{\rm TE}$	$E^0_{\rm TE3}$	$\Delta^{\rm 6D}_{\rm TE3}$	$E^0_{\rm av}$	$\Delta^{\rm 6D}_{\rm av}$
0	*f*	1	5.7036	5.5549	-0.1487	5.7083	0.0047	5.7033	-0.0003	5.7022	-0.0014	5.7009	-0.0027
0	*f*	2	18.9930	18.7392	-0.2538	19.0280	0.0350	18.9921	-0.0009	18.9906	-0.0024	18.9904	-0.0026
0	*f*	3	24.4935	23.7888	-0.7047	24.5912	0.0977	24.4881	-0.0054	24.4857	-0.0078	24.4865	-0.0070
0	*e*	1	0.3830	0.4970	0.1140	0.3923	0.0093	0.3830	0.0000	0.3841	0.0011	0.3858	0.0028
0	*e*	2	1.1553	1.3575	0.2022	1.1454	-0.0099	1.1561	0.0008	1.1569	0.0016	1.1568	0.0015
0	*e*	3	4.3185	4.3790	0.0605	4.3265	0.0080	4.3192	0.0007	4.3199	0.0014	4.3212	0.0027
0	*e*	4	13.0607	13.0760	0.0153	13.0709	0.0102	13.0617	0.0010	13.0622	0.0015	13.0633	0.0026
0	*e*	5	18.8875	18.6708	-0.2167	18.9218	0.0343	18.8870	-0.0005	18.8860	-0.0015	18.8862	-0.0013
1	*f*	1	0.8180	0.8217	0.0037	0.8189	0.0009	0.8183	0.0003	0.8179	-0.0001	0.8170	-0.0010
1	*f*	2	4.3429	4.3156	-0.0273	4.3503	0.0074	4.3428	-0.0001	4.3429	0.0000	4.3431	0.0002
1	*f*	3	7.5826	7.4865	-0.0961	7.5916	0.0090	7.5823	-0.0003	7.5815	-0.0011	7.5807	-0.0019
1	*f*	4	11.5520	11.5053	-0.0467	11.5497	-0.0023	11.5527	0.0007	11.5521	0.0001	11.5511	-0.0009
1	*f*	5	13.2637	13.1112	-0.1525	13.2781	0.0144	13.2642	0.0005	13.2642	0.0005	13.2651	0.0014
1	*f*	6	13.5720	13.3975	-0.1745	13.6113	0.0393	13.5711	-0.0009	13.5697	-0.0023	13.5698	-0.0022
1	*f*	7	19.4397	19.0412	-0.3985	19.5007	0.0610	19.4372	-0.0025	19.4356	-0.0041	19.4362	-0.0035
1	*f*	8	20.6379	20.3626*	-0.2753	20.6977	0.0598	20.6353	-0.0026	20.6334	-0.0045	20.6336	-0.0043
1	*e*	1	0.0000	0.0000	0.0000	0.0000	0.0000	0.0000	0.0000	0.0000	0.0000	0.0000	0.0000
1	*e*	2	1.6766	1.8975	0.2209	1.7088	0.0322	1.6777	0.0011	1.6785	0.0019	1.6792	0.0026
1	*e*	3	2.0260	2.1350	0.1090	1.9982	-0.0278	2.0258	-0.0002	2.0266	0.0006	2.0271	0.0011
1	*e*	4	4.8512	4.8921	0.0409	4.8568	0.0056	4.8514	0.0002	4.8519	0.0007	4.8527	0.0015
1	*e*	5	5.5737	5.6036	0.0299	5.5865	0.0128	5.5737	0.0000	5.5742	0.0005	5.5754	0.0017
1	*e*	6	11.5725	11.5121	-0.0604	11.5739	0.0014	11.5734	0.0009	11.5727	0.0002	11.5717	-0.0008
1	*e*	7	12.3687	12.3498	-0.0189	12.3739	0.0052	12.3690	0.0003	12.3696	0.0009	12.3709	0.0022
1	*e*	8	14.1146	13.8817	-0.2329	14.1549	0.0403	14.1135	-0.0011	14.1122	-0.0024	14.1125	-0.0021
1	*e*	9	15.0844	15.0577	-0.0267	15.1019	0.0175	15.0849	0.0005	15.0850	0.0006	15.0861	0.0017
1	*e*	10	18.4510	18.1310	-0.3200	18.4959	0.0449	18.4499	-0.0011	18.4483	-0.0027	18.4486	-0.0024
1	*e*	11	20.3216	19.7214	-0.6002	20.4091	0.0875	20.3171	-0.0045	20.3151	-0.0065	20.3161	-0.0055
2	*f*	1	2.9509	2.9586	0.0077	2.9550	0.0041	2.9509	0.0000	2.9506	-0.0003	2.9498	-0.0011
2	*f*	2	4.2139	4.1987	-0.0152	4.2154	0.0015	4.2144	0.0005	4.2139	0.0000	4.2129	-0.0010
2	*f*	3	5.5138	5.4753	-0.0385	5.5235	0.0097	5.5135	-0.0003	5.5134	-0.0004	5.5135	-0.0003
2	*f*	4	10.5843	10.5140	-0.0703	10.6004	0.0161	10.5837	-0.0006	10.5831	-0.0012	10.5827	-0.0016
2	*f*	5	11.5880	11.5705	-0.0175	11.5898	0.0018	11.5885	0.0005	11.5885	0.0005	11.5885	0.0005
2	*f*	6	14.2711	14.2137	-0.0574	14.2829	0.0118	14.2715	0.0004	14.2709	-0.0002	14.2705	-0.0006
2	*f*	7	15.3448	15.2310	-0.1138	15.3636	0.0188	15.3450	0.0002	15.3448	0.0000	15.3456	0.0008
2	*f*	8	15.8112	15.5999	-0.2113	15.8485	0.0373	15.8100	-0.0012	15.8087	-0.0025	15.8090	-0.0022
2	*f*	9	19.4950	19.1463	-0.3487	19.5415	0.0465	19.4936	-0.0014	19.4921	-0.0029	19.4926	-0.0024
2	*e*	1	1.2517	1.2390	-0.0127	1.2543	0.0026	1.2516	-0.0001	1.2516	-0.0001	1.2517	0.0000
2	*e*	2	2.6940	2.8312	0.1372	2.7051	0.0111	2.6947	0.0007	2.6953	0.0013	2.6957	0.0017
2	*e*	3	4.1481	4.3089	0.1608	4.1600	0.0119	4.1487	0.0006	4.1494	0.0013	4.1499	0.0018
2	*e*	4	4.6762	4.7326	0.0564	4.6669	-0.0093	4.6766	0.0004	4.6765	0.0003	4.6758	-0.0004
2	*e*	5	6.6583	6.7162	0.0579	6.6638	0.0055	6.6587	0.0004	6.6591	0.0008	6.6596	0.0013
2	*e*	6	7.7316	7.7432	0.0116	7.7502	0.0186	7.7316	0.0000	7.7318	0.0002	7.7328	0.0012
2	*e*	7	12.1600	12.1417	-0.0183	12.1634	0.0034	12.1604	0.0004	12.1606	0.0006	12.1607	0.0007
2	*e*	8	12.8994	12.8903	-0.0091	12.9081	0.0087	12.9005	0.0011	12.9005	0.0011	12.9008	0.0014
2	*e*	9	14.3625	14.2441	-0.1184	14.3773	0.0148	14.3623	-0.0002	14.3618	-0.0007	14.3620	-0.0005
2	*e*	10	17.1130	16.8940	-0.2190	17.1566	0.0436	17.1118	-0.0012	17.1106	-0.0024	17.1109	-0.0021
2	*e*	11	17.9222	17.8356	-0.0866	17.9493	0.0271	17.9221	-0.0001	17.9219	-0.0003	17.9228	0.0006
2	*e*	12	19.5195	19.2640	-0.2555	19.5571	0.0376	19.5189	-0.0006	19.5176	-0.0019	19.5176	-0.0019

TABLE SVII. *Continuation*

J	P	$n_{J,P}$	$E^0_{6\mathrm{D}}$	$E^0_{r_\mathrm{e}}$	$\Delta^{6\mathrm{D}}_{r_\mathrm{e}}$	$E^0_{\langle r\rangle}$	$\Delta^{6\mathrm{D}}_{\langle r\rangle}$	E^0_{TE}	$\Delta^{6\mathrm{D}}_{\mathrm{TE}}$	E^0_{TE3}	$\Delta^{6\mathrm{D}}_{\mathrm{TE3}}$	E^0_{av}	$\Delta^{6\mathrm{D}}_{\mathrm{av}}$
3	f	1	6.0948	6.1092	0.0144	6.1040	0.0092	6.0947	-0.0001	6.0944	-0.0004	6.0936	-0.0012
3	f	2	6.9058	6.8736	-0.0322	6.9132	0.0074	6.9061	0.0003	6.9056	-0.0002	6.9047	-0.0011
3	f	3	7.7150	7.6748	-0.0402	7.7282	0.0132	7.7147	-0.0003	7.7144	-0.0006	7.7142	-0.0008
3	f	4	11.8751	11.8517	-0.0234	11.8762	0.0011	11.8763	0.0012	11.8757	0.0006	11.8745	-0.0006
3	f	5	12.9707	12.9461	-0.0246	12.9812	0.0105	12.9712	0.0005	12.9711	0.0004	12.9710	0.0003
3	f	6	14.5630	14.5164	-0.0466	14.5820	0.0190	14.5623	-0.0007	14.5618	-0.0012	14.5614	-0.0016
3	f	7	17.4866	17.4254	-0.0612	17.5052	0.0186	17.4869	0.0003	17.4862	-0.0004	17.4857	-0.0009
3	f	8	18.4278	18.3288	-0.0990	18.4525	0.0247	18.4277	-0.0001	18.4274	-0.0004	18.4280	0.0002
3	f	9	19.0276	18.8065	-0.2211	19.0661	0.0385	19.0263	-0.0013	19.0251	-0.0025	19.0253	-0.0023
3	e	1	3.4958	3.4734	-0.0224	3.5034	0.0076	3.4954	-0.0004	3.4954	-0.0004	3.4956	-0.0002
3	e	2	4.3480	4.4478	0.0998	4.3620	0.0140	4.3485	0.0005	4.3489	0.0009	4.3495	0.0015
3	e	3	6.8389	7.0479	0.2090	6.8568	0.0179	6.8395	0.0006	6.8404	0.0015	6.8412	0.0023
3	e	4	7.9389	8.0058	0.0669	7.9343	-0.0046	7.9390	0.0001	7.9390	0.0001	7.9384	-0.0005
3	e	5	9.4446	9.5266	0.0820	9.4524	0.0078	9.4449	0.0003	9.4452	0.0006	9.4456	0.0010
3	e	6	10.8110	10.8068	-0.0042	10.8366	0.0256	10.8106	-0.0004	10.8107	-0.0003	10.8117	0.0007
3	e	7	11.9389	11.9197	-0.0192	11.9403	0.0014	11.9399	0.0010	11.9394	0.0005	11.9382	-0.0007
3	e	8	14.1731	14.1717	-0.0014	14.1816	0.0085	14.1734	0.0003	14.1736	0.0005	14.1742	0.0011
3	e	9	14.9360	14.9167	-0.0193	14.9469	0.0109	14.9368	0.0008	14.9367	0.0007	14.9369	0.0009
3	e	10	16.9755	16.7698	-0.2057	17.0039	0.0284	16.9747	-0.0008	16.9737	-0.0018	16.9739	-0.0016
4	f	1	10.1623	10.1887	0.0264	10.1791	0.0168	10.1620	-0.0003	10.1616	-0.0007	10.1610	-0.0013
4	f	2	10.4706	10.4247	-0.0459	10.4894	0.0188	10.4703	-0.0003	10.4698	-0.0008	10.4694	-0.0012
4	f	3	10.9619	10.9146	-0.0473	10.9763	0.0144	10.9613	-0.0006	10.9608	-0.0011	10.9606	-0.0013
4	f	4	15.0411	14.9713	-0.0698	15.0501	0.0090	15.0416	0.0005	15.0410	-0.0001	15.0404	-0.0007
4	f	5	15.4598	15.4370	-0.0228	15.4748	0.0150	15.4602	0.0004	15.4598	0.0000	15.4596	-0.0002
4	f	6	19.2933	19.2701	-0.0232	19.3207	0.0274	19.2924	-0.0009	19.2920	-0.0013	19.2918	-0.0015
4	e	1	6.7069	6.6859	-0.0210	6.7303	0.0234	6.7066	-0.0003	6.7066	-0.0003	6.7069	0.0000
4	e	2	6.8224	6.8906	0.0682	6.8342	0.0118	6.8223	-0.0001	6.8226	0.0002	6.8231	0.0007
4	e	3	9.8233	10.0311	0.2078	9.8411	0.0178	9.8241	0.0008	9.8250	0.0017	9.8260	0.0027
4	e	4	12.0219	12.1117	0.0898	12.0248	0.0029	12.0220	0.0001	12.0221	0.0002	12.0218	-0.0001
4	e	5	13.1564	13.2509	0.0945	13.1726	0.0162	13.1566	0.0002	13.1568	0.0004	13.1568	0.0004
4	e	6	14.7962	14.7758	-0.0204	14.8300	0.0338	14.7956	-0.0006	14.7955	-0.0007	14.7962	0.0000
4	e	7	15.4975	15.4709	-0.0266	15.5058	0.0083	15.4982	0.0007	15.4976	0.0001	15.4966	-0.0009
4	e	8	17.2561	17.2882	0.0321	17.2706	0.0145	17.2564	0.0003	17.2567	0.0006	17.2573	0.0012
4	e	9	18.0588	18.0230	-0.0358	18.0746	0.0158	18.0593	0.0005	18.0590	0.0002	18.0589	0.0001
4	e	10	20.4486	20.4857	0.0371	20.4902	0.0416	20.4471	-0.0015	20.4459	-0.0027	20.4461	-0.0025
5	f	1	14.7855	14.7530	-0.0325	14.8271	0.0416	14.7847	-0.0008	14.7844	-0.0011	14.7844	-0.0011
5	f	2	14.9593	14.9739	0.0146	14.9810	0.0217	14.9590	-0.0003	14.9585	-0.0008	14.9579	-0.0014
5	f	3	15.3676	15.3068	-0.0608	15.3805	0.0129	15.3669	-0.0007	15.3663	-0.0013	15.3656	-0.0020
5	f	4	18.5669	18.4938	-0.0731	18.5862	0.0193	18.5668	-0.0001	18.5663	-0.0006	18.5662	-0.0007
5	f	5	19.3693	19.2883	-0.0810	19.3912	0.0219	19.3695	0.0002	19.3687	-0.0006	19.3680	-0.0013
5	e	1	10.1202	10.1889	0.0687	10.1547	0.0345	10.1202	0.0000	10.1205	0.0003	10.1214	0.0012
5	e	2	10.8803	10.8378	-0.0425	10.8999	0.0196	10.8792	-0.0011	10.8789	-0.0014	10.8793	-0.0010
5	e	3	13.1073	13.2713	0.1640	13.1249	0.0176	13.1079	0.0006	13.1086	0.0013	13.1097	0.0024
5	e	4	16.5009	16.6501	0.1492	16.5133	0.0124	16.5012	0.0003	16.5016	0.0007	16.5020	0.0011
5	e	5	17.9889	18.0526	0.0637	18.0161	0.0272	17.9884	-0.0005	17.9882	-0.0007	17.9880	-0.0009
5	e	6	19.6326	19.5909	-0.0417	19.6765	0.0439	19.6315	-0.0011	19.6312	-0.0014	19.6319	-0.0007
5	e	7	20.0881	20.0786*	-0.0095	20.1026	0.0145	20.0883	0.0002	20.0878	-0.0003	20.0872	-0.0009
6	f	1	19.7704	19.7499	-0.0205	19.8261	0.0557	19.7692	-0.0012	19.7687	-0.0017	19.7690	-0.0014
6	f	2	20.3159	20.2641*	-0.0518	20.3475	0.0316	20.3150	-0.0009	20.3143	-0.0016	20.3139	-0.0020
6	e	1	14.2507	14.3135	0.0628	14.2982	0.0475	14.2505	-0.0002	14.2507	0.0000	14.2516	0.0009
6	e	2	15.9107	15.8542	-0.0565	15.9419	0.0312	15.9092	-0.0015	15.9089	-0.0018	15.9092	-0.0015
6	e	3	16.9228	17.0341	0.1113	16.9420	0.0192	16.9231	0.0003	16.9237	0.0009	16.9245	0.0017
7	e	1	19.1021	19.1617	0.0596	19.1662	0.0641	19.1016	-0.0005	19.1016	-0.0005	19.1028	0.0007
RMSE					0.1513		0.0276		0.0010		0.0016		0.0018
Max. error					-0.7047		0.0977		-0.0054		-0.0078		-0.0070

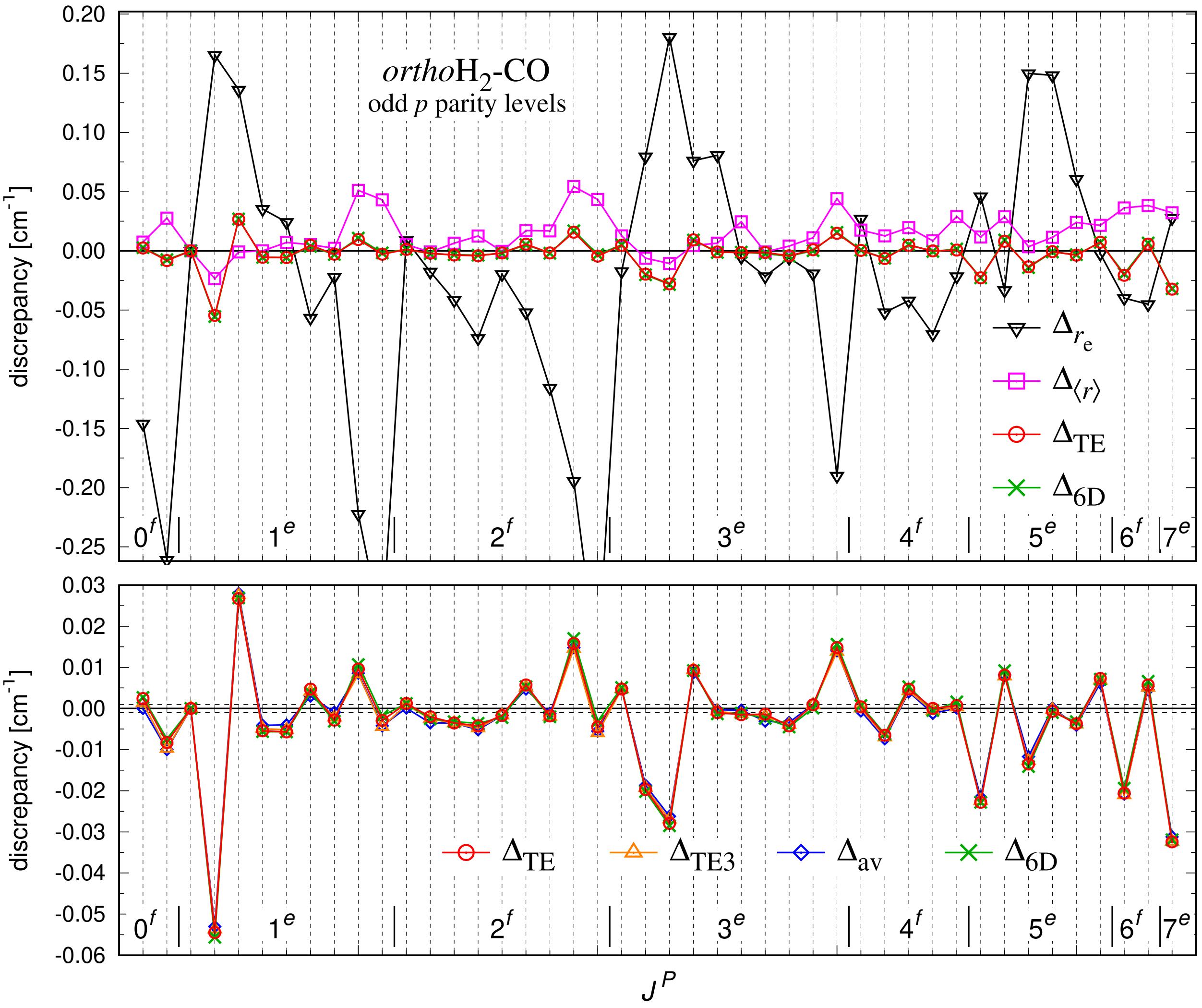

FIG. S2. Comparison of the odd p parity bound rovibrational energy levels of *ortho*H_2–CO with CO in the ground vibrational state, $v_2 = 0$. The figures show the errors of the levels computed using a given method with respect to the experimental levels: $\Delta_{\text{method}} = E^{v_2}_{\text{method}} - E^{v_2}_{\text{expt}}$. The levels on the horizontal axis are ordered according to their ascending value within each J^P symmetry block (see Sec. SV of SI for spectroscopic notation). The lacking entries correspond to the levels for which experimental values were not determined [27]. The numerical values of the energies are listed in Table SVI of SI.

TABLE SVIII. The rovibrational energy levels for *ortho*D_2–CO obtained from the rigid-rotor calculations, $E^0_{r_e}$, $E^0_{\langle r\rangle}$, E^0_{TE}, E^0_{TE3}, and E^0_{av}, using five reduced-dimensionality surfaces: $V(r_{1e}, r_{2e})$, $V(\langle r_1\rangle_0, \langle r_2\rangle_0)$, $\langle V\rangle^{\mathrm{TE}}_{00}$, $\langle V\rangle^{\mathrm{TE3}}_{00}$, and $\langle V\rangle_{00}$, respectively. The values of these energies are compared with the experimental ones [35], E^0_{expt}, using the difference $\Delta_{\mathrm{method}} = E^0_{\mathrm{method}} - E^0_{\mathrm{expt}}$. The calculated energy levels are given relative to the ground level, $(J, P, n_{J,P}) = (0, e, 1)$, that is -25.0253 cm^{-1}, -25.6161 cm^{-1}, -25.5253 cm^{-1}, -25.5241 cm^{-1}, and -25.5248 cm^{-1} for $E^0_{r_e}$, $E^0_{\langle r\rangle}$, E^0_{TE}, E^0_{TE3}, and E^0_{av}, respectively, where the zero of energy is defined by D_2 and CO at infinite separation and in their ground states: $v_1 = v_2 = j_1 = j_2 = 0$. Only the bound states for $v_2 = 0$ are presented in this comparison, except for the two states marked with asterisks, which, in the case of the $V(r_{1e}, r_{2e})$ surface, shallower than the other surfaces, become the resonances. $n_{J,P}$ numbers consecutive energy levels. The values of Δ_{r_e}, $\Delta_{\langle r\rangle}$, Δ_{TE}, Δ_{TE3}, and Δ_{av} are plotted in Fig. 2C. Energies are reported as wavenumbers in cm^{-1}.

J	P	$n_{J,P}$	E^0_{expt}	$E^0_{r_e}$	Δ_{r_e}	$E^0_{\langle r\rangle}$	$\Delta_{\langle r\rangle}$	E^0_{TE}	Δ_{TE}	E^0_{TE3}	Δ_{TE3}	E^0_{av}	Δ_{av}
0	e	1	0.0000	0.0000	0.0000	0.0000	0.0000	0.0000	0.0000	0.0000	0.0000	0.0000	0.0000
0	e	2	6.9781	6.9903	0.0122	7.0068	0.0287	6.9811	0.0030	6.9810	0.0029	6.9817	0.0036
0	e	3	14.0077	13.9848	-0.0229	14.0243	0.0166	14.0016	-0.0061	14.0016	-0.0061	14.0022	-0.0055
0	e	4		22.7445		23.1389		23.0663		23.0655		23.0662	
0	e	5		24.6802		24.8929		24.8445		24.8441		24.8446	
1	f	1	3.5937	3.5995	0.0058	3.5858	-0.0079	3.5854	-0.0083	3.5854	-0.0083	3.5853	-0.0084
1	f	2	14.5896	14.6071	0.0175	14.6133	0.0237	14.5900	0.0004	14.5900	0.0004	14.5904	0.0008
1	f	3		24.0312		24.2104		24.1685		24.1681		24.1684	
1	f	4		28.4770		28.8242		28.7600		28.7593		28.7600	
1	e	1	0.6090	0.6111	0.0021	0.6107	0.0017	0.6099	0.0009	0.6099	0.0009	0.6099	0.0009
1	e	2	3.4289	3.4335	0.0046	3.4208	-0.0081	3.4198	-0.0091	3.4198	-0.0091	3.4197	-0.0092
1	e	3	7.6870	7.6998	0.0128	7.7153	0.0283	7.6895	0.0025	7.6895	0.0025	7.6901	0.0031
1	e	4	13.3297	13.3281	-0.0016	13.3498	0.0201	13.3277	-0.0020	13.3277	-0.0020	13.3282	-0.0015
1	e	5	15.7809	15.7786	-0.0023	15.8043	0.0234	15.7797	-0.0012	15.7796	-0.0013	15.7801	-0.0008
1	e	6		23.0576		23.3615		23.3116		23.3110		23.3116	
1	e	7		23.3853		23.6199		23.5597		23.5591		23.5596	
2	f	1	4.8480	4.8562	0.0082	4.8415	-0.0065	4.8394	-0.0086	4.8394	-0.0086	4.8392	-0.0088
2	f	2	11.1002	11.1093	0.0091	11.0899	-0.0103	11.0898	-0.0104	11.0898	-0.0104	11.0896	-0.0106
2	f	3	16.0311	16.0498	0.0187	16.0549	0.0238	16.0311	0.0000	16.0310	-0.0001	16.0315	0.0004
2	f	4	24.4871	24.4100	-0.0771	24.5156	0.0285	24.4843	-0.0028	24.4840	-0.0031	24.4843	-0.0028
2	f	5		26.4848		26.6290		26.5884		26.5881		26.5885	
2	e	1	1.8244	1.8298	0.0054	1.8287	0.0043	1.8263	0.0019	1.8263	0.0019	1.8263	0.0019
2	e	2	4.3710	4.3799	0.0089	4.3678	-0.0032	4.3641	-0.0069	4.3641	-0.0069	4.3640	-0.0070
2	e	3	9.0776	9.0935	0.0159	9.1074	0.0298	9.0811	0.0035	9.0811	0.0035	9.0817	0.0041
2	e	4	11.0234	11.0314	0.0080	11.0142	-0.0092	11.0131	-0.0103	11.0131	-0.0103	11.0129	-0.0105
2	e	5	13.7981	13.7971	-0.0010	13.8161	0.0180	13.7953	-0.0028	13.7952	-0.0029	13.7957	-0.0024
2	e	6	17.8523	17.8480	-0.0043	17.8783	0.0260	17.8512	-0.0011	17.8511	-0.0012	17.8516	-0.0007
2	e	7	23.1787	23.1098	-0.0689	23.2065	0.0278	23.1776	-0.0011	23.1774	-0.0013	23.1778	-0.0009
2	e	8		23.8423		24.2449		24.1694		24.1686		24.1693	
3	f	1	6.7181	6.7321	0.0140	6.7161	-0.0020	6.7113	-0.0068	6.7113	-0.0068	6.7111	-0.0070
3	f	2	12.5831	12.5960	0.0129	12.5772	-0.0059	12.5739	-0.0092	12.5738	-0.0093	12.5737	-0.0094
3	f	3	18.1398	18.1603	0.0205	18.1648	0.0250	18.1396	-0.0002	18.1396	-0.0002	18.1400	0.0002
3	f	4	22.5232	22.5338	0.0106	22.5139	-0.0093	22.5137	-0.0095	22.5137	-0.0095	22.5135	-0.0097
3	f	5	25.7540	25.6767	-0.0773	25.7831	0.0291	25.7518	-0.0022	25.7516	-0.0024	25.7518	-0.0022
3	f	6		28.1034		28.3303		28.2780		28.2775		28.2779	
3	e	1	3.6404	3.6492	0.0088	3.6472	0.0068	3.6425	0.0021	3.6425	0.0021	3.6425	0.0021
3	e	2	5.8200	5.8310	0.0110	5.8194	-0.0006	5.8121	-0.0079	5.8120	-0.0080	5.8120	-0.0080
3	e	3	11.1147	11.1328	0.0181	11.1447	0.0300	11.1174	0.0027	11.1173	0.0026	11.1179	0.0032
3	e	4	12.2536	12.2624	0.0088	12.2521	-0.0015	12.2451	-0.0085	12.2451	-0.0085	12.2451	-0.0085
3	e	5	15.0954	15.0981	0.0027	15.1104	0.0150	15.0916	-0.0038	15.0916	-0.0038	15.0920	-0.0034
3	e	6	20.4133	20.4011	-0.0122	20.4448	0.0315	20.4137	0.0004	20.4135	0.0002	20.4140	0.0007
3	e	7		22.3857		22.3769		22.3733		22.3733		22.3731	
3	e	8	23.8343	23.7871	-0.0472	23.8532	0.0189	23.8306	-0.0037	23.8304	-0.0039	23.8307	-0.0036
3	e	9		24.8393		25.2651		25.1872		25.1863		25.1870	

J	P	$n_{J,P}$	E^0_{expt}	$E^0_{r_\mathrm{e}}$	Δ_{r_e}	$E^0_{\langle r\rangle}$	$\Delta_{\langle r\rangle}$	E^0_{TE}	Δ_{TE}	E^0_{TE3}	Δ_{TE3}	E^0_{av}	Δ_{av}
4	f	1	9.1965	9.2156	0.0191	9.1982	0.0017	9.1898	-0.0067	9.1898	-0.0067	9.1896	-0.0069
4	f	2	14.6018	14.6193	0.0175	14.6012	-0.0006	14.5939	-0.0079	14.5939	-0.0079	14.5938	-0.0080
4	f	3	20.8746	20.8969	0.0223	20.9020	0.0274	20.8746	0.0000	20.8745	-0.0001	20.8749	0.0003
4	f	4	24.1343	24.1472	0.0129	24.1320	-0.0023	24.1266	-0.0077	24.1266	-0.0077	24.1265	-0.0078
4	f	5	27.7011	27.5945	-0.1066	27.7352	0.0341	27.6987	-0.0024	27.6984	-0.0027	27.6986	-0.0025
4	e	1	6.0446	6.0587	0.0141	6.0558	0.0112	6.0480	0.0034	6.0479	0.0033	6.0480	0.0034
4	e	2	7.7855	7.8025	0.0170	7.7912	0.0057	7.7795	-0.0060	7.7795	-0.0060	7.7795	-0.0060
4	e	3	13.7028	13.7366	0.0338	13.7296	0.0268	13.7098	0.0070	13.7097	0.0069	13.7100	0.0072
4	e	4	13.8802	13.8785	-0.0017	13.8938	0.0136	13.8711	-0.0091	13.8710	-0.0092	13.8714	-0.0088
4	e	5	17.2012	17.2102	0.0090	17.2148	0.0136	17.1972	-0.0040	17.1971	-0.0041	17.1974	-0.0038
4	e	6	23.3630	23.3225	-0.0405	23.4027	0.0397	23.3659	0.0029	23.3657	0.0027	23.3661	0.0031
4	e	7	23.5302	23.5199	-0.0103	23.5414	0.0112	23.5261	-0.0041	23.5260	-0.0042	23.5262	-0.0040
4	e	8	25.4637	25.4341*	-0.0296	25.4775	0.0138	25.4597	-0.0040	25.4596	-0.0041	25.4596	-0.0041
5	f	1	12.2636	12.2899	0.0263	12.2715	0.0079	12.2584	-0.0052	12.2584	-0.0052	12.2583	-0.0053
5	f	2	17.1632	17.1865	0.0233	17.1691	0.0059	17.1575	-0.0057	17.1575	-0.0057	17.1574	-0.0058
5	f	3	24.1935	24.2171	0.0236	24.2255	0.0320	24.1949	0.0014	24.1948	0.0013	24.1951	0.0016
5	f	4	26.2051	26.2221	0.0170	26.2108	0.0057	26.2004	-0.0047	26.2003	-0.0048	26.2003	-0.0048
5	e	1	9.0234	9.0431	0.0197	9.0399	0.0165	9.0280	0.0046	9.0280	0.0046	9.0280	0.0046
5	e	2	10.2774	10.2988	0.0214	10.2879	0.0105	10.2709	-0.0065	10.2709	-0.0065	10.2710	-0.0064
5	e	3	15.7948	15.8107	0.0159	15.8097	0.0149	15.7925	-0.0023	15.7924	-0.0024	15.7927	-0.0021
5	e	4	16.9873	17.0060	0.0187	17.0222	0.0349	16.9885	0.0012	16.9884	0.0011	16.9890	0.0017
5	e	5	20.0265	20.0428	0.0163	20.0419	0.0154	20.0233	-0.0032	20.0232	-0.0033	20.0233	-0.0032
5	e	6	24.9734	24.9685	-0.0049	24.9894	0.0160	24.9712	-0.0022	24.9711	-0.0023	24.9714	-0.0020
6	f	1	15.8988	15.9317	0.0329	15.9134	0.0146	15.8946	-0.0042	15.8945	-0.0043	15.8944	-0.0044
6	f	2	20.2658	20.2942	0.0284	20.2785	0.0127	20.2619	-0.0039	20.2618	-0.0040	20.2618	-0.0040
6	f	3	28.0480	28.0647	0.0167	28.0846	0.0366	28.0497	0.0017	28.0496	0.0016	28.0499	0.0019
6	f	4	28.7785	28.8020	0.0235	28.7910	0.0125	28.7756	-0.0029	28.7755	-0.0030	28.7755	-0.0030
6	e	1	12.5563	12.5824	0.0261	12.5800	0.0237	12.5631	0.0068	12.5631	0.0068	12.5631	0.0068
6	e	2	13.2853	13.3125	0.0272	13.3027	0.0174	13.2795	-0.0058	13.2795	-0.0058	13.2796	-0.0057
6	e	3	18.2491	18.2664	0.0173	18.2708	0.0217	18.2488	-0.0003	18.2487	-0.0004	18.2491	0.0000
6	e	4	20.7262	20.7474	0.0212	20.7663	0.0401	20.7289	0.0027	20.7287	0.0025	20.7293	0.0031
6	e	5	23.4882	23.5120	0.0238	23.5093	0.0211	23.4875	-0.0007	23.4874	-0.0008	23.4875	-0.0007
7	f	1	20.0690	20.1087	0.0397	20.0925	0.0235	20.0667	-0.0023	20.0666	-0.0024	20.0665	-0.0025
7	f	2	23.8969	23.9309	0.0340	23.9182	0.0213	23.8961	-0.0008	23.8961	-0.0008	23.8961	-0.0008
7	e	1	16.6197	16.6495	0.0298	16.6502	0.0305	16.6270	0.0073	16.6269	0.0072	16.6270	0.0073
7	e	2	16.7932	16.8246	0.0314	16.8167	0.0235	16.7868	-0.0064	16.7867	-0.0065	16.7869	-0.0063
7	e	3	21.1936	21.2126	0.0190	21.2216	0.0280	21.1953	0.0017	21.1952	0.0016	21.1956	0.0020
7	e	4	24.9490	24.9647	0.0157	24.9913	0.0423	24.9491	0.0001	24.9490	0.0000	24.9494	0.0004
8	f	1	24.7351	24.7743	0.0392	24.7639	0.0288	24.7295	-0.0056	24.7294	-0.0057	24.7294	-0.0057
8	f	2	28.0409	28.0776	0.0367	28.0704	0.0295	28.0424	0.0015	28.0423	0.0014	28.0424	0.0015
8	e	1	20.7643	20.7977	0.0334	20.7965	0.0322	20.7568	-0.0075	20.7566	-0.0077	20.7570	-0.0073
8	e	2	21.1767	21.2106	0.0339	21.2161	0.0394	21.1870	0.0103	21.1869	0.0102	21.1871	0.0104
8	e	3	24.6331	24.6550	0.0219	24.6688	0.0357	24.6385	0.0054	24.6384	0.0053	24.6389	0.0058
9	e	1	25.1512	25.1786*	0.0274	25.1880	0.0368	25.1383	-0.0129	25.1381	-0.0131	25.1385	-0.0127

TABLE SIX. A comparison of energies of resonances for *ortho*H_2–CO (the $v_2 = 0$ case) calculated from various 4D surfaces with the corresponding experimental values $E^0_{\rm expt}$ [27]. The energies denoted by $E^0_{r_e}$, $E^0_{\langle r\rangle}$, $E^0_{\rm TE}$, $E^0_{\rm TE3}$, and $E^0_{\rm av}$, were obtained using the $V(r_{1e}, r_{2e})$, $V(\langle r_1\rangle_0, \langle r_2\rangle_0)$, $\langle V\rangle^{\rm TE}_{00}$, $\langle V\rangle^{\rm TE3}_{00}$, and $\langle V\rangle_{00}$ surfaces, respectively, The calculated even-parity (odd-parity) energy levels are given relative to the $(J, P, n_{J,P}) = (1, f, 1)$ $((1, e, 1))$ level, and their values are given in the caption to Table SV (SVI). The values of the theoretical energies are compared with the experimental ones [27], $E^0_{\rm expt}$, using the difference $\Delta_{\rm method} = E^0_{\rm method} - E^0_{\rm expt}$. Only the resonances for which the experimental values are known [27] are listed in the table. In the column $n_{J,P}$, we give the consecutive numbers of the states in each block of symmetry J^P, according to the order of states in Tables S2 and S3 from Ref. [27]. The RMSEs are calculated for all resonances from the table. Energies are reported as wavenumbers in cm^{-1}.

J	P	$n_{J,P}$	$E^0_{\rm expt}$	$E^0_{r_e}$	Δ_{r_e}	$E^0_{\langle r\rangle}$	$\Delta_{\langle r\rangle}$	$E^0_{\rm TE}$	$\Delta_{\rm TE}$	$E^0_{\rm TE3}$	$\Delta_{\rm TE3}$	$E^0_{\rm av}$	$\Delta_{\rm av}$
						even-parity states							
5	f	6	23.5258	23.5198	-0.0060	23.5489	0.0231	23.5193	-0.0065	23.5194	-0.0064	23.5202	-0.0056
6	e	4	20.3407	20.5062	0.1655	20.3513	0.0106	20.3311	-0.0096	20.3321	-0.0086	20.3336	-0.0071
6	e	5	23.0611	23.0833	0.0222	23.0993	0.0382	23.0589	-0.0022	23.0588	-0.0023	23.0594	-0.0017
6	e	6	24.5332	24.5721	0.0389	24.5627	0.0295	24.5424	0.0092	24.5426	0.0094	24.5434	0.0102
6	e	7	25.5226	25.5451	0.0225	25.5533	0.0307	25.5220	-0.0006	25.5222	-0.0004	25.5230	0.0004
7	f	1	24.5420	24.4791	-0.0629	24.5901	0.0481	24.5186	-0.0234	24.5184	-0.0236	24.5197	-0.0223
7	f	2	25.4672	25.3551	-0.1121	25.5212	0.0540	25.4719	0.0047	25.4714	0.0042	25.4719	0.0047
8	e	1	23.7238	23.7214	-0.0024	23.7642	0.0404	23.6835	-0.0403	23.6837	-0.0401	23.6860	-0.0378
8	e	2	25.9887	25.9975	0.0088	26.0327	0.0440	26.0015	0.0128	26.0018	0.0131	26.0033	0.0146
8	e	3	30.5061	30.6238	0.1177	30.5284	0.0223	30.4956	-0.0105	30.4964	-0.0097	30.4980	-0.0081
						odd-parity states							
3	e	11	21.0651	20.8640	-0.2011	21.1248	0.0597	21.0738	0.0087	21.0727	0.0076	21.0730	0.0079
3	e	12	24.4049	24.2823	-0.1226	24.4706	0.0657	24.4097	0.0048	24.4083	0.0034	24.4084	0.0035
4	f	7	21.5966	21.5190	-0.0776	21.6304	0.0338	21.6026	0.0060	21.6018	0.0052	21.6014	0.0048
4	f	10	25.0009	24.8919	-0.1090	25.0584	0.0575	25.0361	0.0352	25.0355	0.0346	25.0356	0.0347
5	e	8	21.3003	21.3578	0.0575	21.3212	0.0209	21.3003	0.0000	21.3005	0.0002	21.3011	0.0008
5	e	9	22.2227	22.1735	-0.0492	22.2502	0.0275	22.2265	0.0038	22.2260	0.0033	22.2259	0.0032
6	f	3	20.8166	20.7961	-0.0205	20.8566	0.0400	20.8343	0.0177	20.8337	0.0171	20.8334	0.0168
6	f	4	22.8940	22.8149	-0.0791	22.9277	0.0337	22.8989	0.0049	22.8982	0.0042	22.8980	0.0040
6	f	5	24.2263	24.0758	-0.1505	24.2740	0.0477	24.2365	0.0102	24.2354	0.0091	24.2349	0.0086
7	e	2	21.4539	21.5121	0.0582	21.4845	0.0306	21.4622	0.0083	21.4624	0.0085	21.4634	0.0095
7	e	3	21.6813	21.6065	-0.0748	21.7366	0.0553	21.6882	0.0069	21.6876	0.0063	21.6879	0.0066
7	e	4	26.0644	26.2230	0.1586	26.0821	0.0177	26.0550	-0.0094	26.0555	-0.0089	26.0565	-0.0079
RMSE					0.0961		0.0405		0.0148		0.0144		0.0140
Max error					-0.2011		0.0657		-0.0403		-0.0401		-0.0378

TABLE SX. Comparison of RMSEs (in cm^{-1}) of rovibrational energy levels or transition energies from reduced and full-dimensionality PESs relative to experimental values. Energy levels are given relative to the dimer ground-state level. Monomers are in their ground rovibrational states. For Ar–HF, all levels listed in Table VI of Ref. [36] are included, while for $(H_2O)_2$, only the $J = 0$ levels. In the case of Ref. [11], transition energies listed in Table 1 of that work are used. In the case of Ref. [13], the RMSEs are computed from band origins and tunneling splittings in the same way as Table 17 of Ref. [37]. The $\langle V \rangle$ results are taken from $N_v = 1$ results of Ref. [13]. In the Diff column, the relative differences of RMSEs with respect to the 3D (Ar–HF) and 6D+6D or 12D ($(H_2O)_2$) values are listed.

Dimer	Dimension	Reduction	RMSE	Diff	Ref.
Ar–HF	2D	$V(r_\mathrm{e})$	4.92	34.6%	[36]
		$V(\langle r \rangle)$	4.12	12.7%	
		$\langle V \rangle$	3.67	0.5%	
	3D	none	3.65		
$(H_2O)_2$	6D	$V(\langle r \rangle)$	7.91	6.9%	[11]
	6D+6D	adiabatic	7.40		
$(H_2O)_2$	6D	$V(r_\mathrm{e})$	1.94	15.5%	[13]
		$V(\langle r \rangle)$	1.21	-28.0%	[15, 38]
		$\langle V \rangle$	1.71	1.9%	[13]
	12D	none	1.68		[13]

TABLE SXI. Basis set parameters for H_2–CO. N_{r_0} is the number of sine DVR basis functions. N_{r_1} and N_{r_2} are the numbers of PODVR basis functions. N_{θ_i} and N_ϕ are the numbers of θ_i and ϕ quadrature points. $N_{\rm bas}$ is the number of $J = 0$ even-parity basis functions for *para*H_2–CO. Upper-line ranges of r_1 and r_2 were used for probing isolated monomer potentials in order to obtain the PODVR bases, lower-line ranges were used for probing the H_2–CO 6D potential energy surface U_{23}. Distances are in bohr.

Basis	N_{r0}	N_{r_1}	N_{r_2}	$\max(j_1, m_1)$	$\max(j_2, m_2)$	$(N_{\theta_1}, N_{\theta_2}, N_\phi)$	$N_{\rm bas}$(4D)	$N_{\rm bas}$
	$[r_{0\rm min}, r_{0\rm max}]$	$[r_{1\rm min}, r_{1\rm max}]$	$[r_{2\rm min}, r_{2\rm max}]$					
Basis I	180	3	5	14	14	(15,15,30)	117K	1760K
	[4.0, 80.0]	[0.40, 13.00]	[1.75, 2.85]					
		[1.24, 1.86][a]	[1.99, 2.36][b]					
Basis II	80	3	5	3	10	(6,11,22)	3280	49.2K
	[4.0, 50.0]	[0.40, 13.00]	[1.75, 2.85]					
		[1.24, 1.86][a]	[1.99, 2.36][b]					
Basis III	80	5	5	3	10	(6,11,22)	3280	82.0K
	[4.0, 50.0]	[0.40, 13.00]	[1.75, 2.85]					
		[1.14, 2.21][c]	[1.99, 2.36][b]					

[a] The values of r_1 PODVR points used in the DSL calculations: 1.24477, 1.53493, and 1.85929 bohr.
[b] The values of r_2 PODVR points used in the DSL calculations: 1.98877, 2.08115, 2.16786, 2.25785, and 2.36155 bohr.
[c] The values of r_1 PODVR points used in the DSL calculations: 1.14325, 1.38539, 1.62613, 1.88933, and 2.20932 bohr.

[1] T. Zhang, J. Wang, D. Yang, and D. Xie, "Full-Dimensional Time-Independent Quantum Dynamics Approach to Rovibrationally Inelastic Scattering between Triatomic and Diatomic Molecules: A Case Study for $H_2O + H_2$," J. Chem. Theory Comput. **21**, 11365–11376 (2025).

[2] M. Quack and M. A. Suhm, "Potential energy surface and energy levels of $(HF)_2$ and its D isotopomers," Mol. Phys. **69**, 791–801 (1990).

[3] M. Quack and M. A. Suhm, "Potential energy surfaces, qausiadiabatic channels, rovibrational spectra, and intramolecular dynamics of $(HF)_2$ and its isotopomers from quantum monte carlo calculations," J. Chem. Phys. **95**, 28–59 (1991).

[4] D. H. Zhang and J. Z. H. Zhang, "Photofragmentation of HF dimer: Quantum dynamics studies on ab initio potential energy surfaces," J. Chem Phys. **99**, 6624–6633 (1995).

[5] W. C. Necoechea and D. G. Truhlar, "A converged full-dimensional calculation of the vibrational energy levels of $(HF)_2$," Chem. Phys. Lett. **224**, 297–304 (1994).

[6] D. H. Zhang, Q. Wu, J. Z. H. Zhang, M. von Dirke, and Z. Bačič, "Exact full-dimensional bound state calculations for $(HF)_2$, $(DF)_2$ and HFDF," J. Chem Phys. **102**, 2315–2325 (1995).

[7] P. R. Bunker, P. Jensen, A. Karpfen, M. Kofranek, and H. Lischka, "An ab initio calculation of the stretching energies for the hf dimer," J. Chem. Phys. **92**, 7432–7440 (1990).

[8] J. Huang, D. Yang, Y. Zhou, and D. Xie, "A new full-dimensional *ab initio* intermolecular potential energy surface and vibrational states for $(HF)_2$ and $(DF)_2$," J. Chem. Phys. **150**, 154302 (2019).

[9] P. M. Felker and Z. Bačić, "Weakly bound molecular dimers: Intramolecular vibrational fundamentals, overtones, and tunneling splittings from full-dimensional quantum calculations using compact contracted bases of intramolecular and low-energy rigid-monomer intermolecular eigenstates," J. Chem. Phys. **151**, 024305 (2019).

[10] R. I. Ovsyannikov, V. Y. Makhnev, N. F. Zobov, J. Koput, J. Tennyson, and O. L. Polyansky, "Highly accurate hf dimer *ab initio* potential energy surface," J. Chem. Phys. **156**, 164305 (2022).

[11] K. Szalewicz, G. Murdachaew, R. Bukowski, O. Akin-Ojo, and C. Leforestier, "Spectra of water dimer from *ab initio* calculations," in *Lecture Series on Computer and Computational Science: ICCMSE 2006*, Vol. 6, edited by G. Maroulis and T. Simos (Brill Academic Publish-

ers, Leiden, 2006) pp. 482–491.

[12] C. Leforestier, F. Gatti, R. S. Fellers, and R. J. Saykally, "Determination of a flexible (12d) water dimer potential via direct inversion of spectroscopic data," J. Phys. Chem. **117**, 8710–8722 (2002).

[13] X.-G. Wang and T. Carrington, Jr., "Using monomer vibrational wavefunctions to compute numerically exact (12D) rovibrational levels of water dimer," J. Chem. Phys. **148**, 074108 (2018).

[14] C. Leforestier, "Infrared shifts of the water dimer from the fully flexible *ab initio* HBB2 potential," Philos. Trans. R. Soc. A **370**, 2675–2690 (2012).

[15] C. Leforestier, K. Szalewicz, and A. van der Avoird, "Spectra of water dimer from a new *ab initio* potential with flexible monomers," J. Chem. Phys. **137**, 014305 (2012).

[16] V. Babin, C. Leforestier, and F. Paesani, "Development of a "First Principles" water potential with flexible monomers: Dimer potential energy surface, VRT spectrum, and second virial coefficient," J. Chem. Theory Comput. **9**, 5395–5403 (2013).

[17] X.-G. Wang and T. Carrington, Jr., "Computing excited oh stretch states of water dimer in 12d using contracted intermolecular and intramolecular basis functions," J. Chem. Phys. **158**, 084107 (2023).

[18] S. K. Pogrebnya and D. C. Clary, "A full-dimensional quantum dynamical study of vibrational relaxation in $H_2 + H_2$," Chem. Phys. Lett. **363**, 523–526 (2002).

[19] A. I. Boothroyd, P. G. Martin, W. J. Keogh, and M. J. Peterson, "An accurate analytic H_4 potential energy surface," J. Chem. Phys. **116**, 666–689 (2002).

[20] B. Yang, P. Zhang, X. Wang, P. C. Stancil, J. M. Bowman, N. Balakrishnan, and R. C. Forrey, "Quantum dynamics of CO–H_2 in full dimensionality," Nature Comm. **6**, 6629 (2015).

[21] B. Yang, K. M. Walker, P. Forrey, P. C. Stancil, and N. Balakrishnan, "Collisional quenching of highly rotationally excited HF," AA **578**, A65 (2015).

[22] A. Faure, P. Jankowski, T. Stoecklin, and K. Szalewicz, "On the importance of full-dimensionality in low-energy molecular scattering calculations," Sci. Rep. (Nature) **6**, 28449 (2016).

[23] C. Castro, K. Doan, M. Klamka, R. C. Forrey, B. Yang, P. C. Stancil, and N. Balakrishnan, "Inelastic cross sections and rate coefficients for collisions between CO and H_2," Mol. Astrophys. **6**, 47–58 (2017).

[24] Q. Yao, M. Morita, C. Xie, N. Balakrishnan, and H. Guo, "Globally accurate full-dimensional potential energy surface for H_2 + HCl inelastic scattering," J. Phys. Chem. A **123**, 6578–6586 (2019).

[25] N. Balakrishnan, P. G. Jambrina, J. F. E. Croft, H. Guo, and F. J. Aoiz, "Quantum stereodynamics of cold molecular collisions," Chem. Comm. **60**, 1239–1256 (2024).

[26] B. Mandal, J. F. E. Croft, P. G. Jambrina, H. Guo, F. J. Aoiz, and N. Balakrishnan, "Stereodynamic control of cold rotationally inelastic HD + D_2 collisions," Phys. Chem. Chem. Phys. **26**, 18368–18381 (2024).

[27] M. Stachowiak, E. Grabowska, X.-G. Wang, T. Carrington, Jr., K. Szalewicz, and P. Jankowski, "Theory cracks old data: rovibrational energy levels of *ortho*H_2–CO derived from experiment," Sci. Adv. **10**, eadj8632 (2024).

[28] R. A. Kendall, T. H. Dunning, Jr., and R. J. Harrison, "Electron affinities of the first-row atoms revisited. Systematic basis sets and wave functions," J. Chem Phys. **96**, 6796–6806 (1992).

[29] B. Fernández, H. Koch, and J. Makarewicz, "Accurate intermolecular ground state potential of the Ar-N_2 complex," J. Chem. Phys. **110**, 8525–8532 (1999).

[30] B. J. Braams and J. M. Bowman, "Permutationally invariant potential energy surfaces in high dimensionality," Intern. Rev. Phys. Chem. **28**, 577–606 (2009).

[31] G. Garberoglio, P. Jankowski, K. Szalewicz, and A. H. Harvey, "All-dimensional H_2-CO potential: Validation with fully quantum second virial coefficients," J. Chem. Phys. **146**, 054304–1,13 (2017).

[32] P. Jankowski and K. Szalewicz, "A new *ab initio* interaction energy surface and high-resolution spectra of the H_2–CO van der Waals complex," J. Chem. Phys. **123**, 104301 (2005).

[33] P. Jankowski, L. A. Surin, A. V. Potapov, S. Schlemmer, A. R. W. McKellar, and K. Szalewicz, "A comprehensive experimental and theoretical study of H_2-CO spectra," J. Chem. Phys. **138**, 084307 (2013).

[34] A. R. W. McKellar, "High-resolution infrared spectrum and energy levels of the weakly bound complex, CO-paraH_2," J. Chem. Phys. **108**, 1811–1820 (1998).

[35] A. R. W. McKellar, "High-resolution infrared spectrum and energy levels of the weakly bound complex, CO-orthoD_2," J. Chem. Phys. **112**, 9282–9288 (2000).

[36] M. Jeziorska, P. Jankowski, K. Szalewicz, and B. Jeziorski, "On the optimal choice of monomer geometry in calculations of intermolecular interaction energies: Rovibrational spectrum of Ar-HF from two- and three-dimensional potentials," J. Chem. Phys. **113**, 2957–2968 (2000).

[37] M. P. Metz, K. Szalewicz, J. Sarka, R. Tóbiás, A. Császár, and E. Matyus, "Molecular dimers of methane clathrates: ab initio potential energy surfaces and variational vibrational states," Phys. Chem. Chem. Phys. **21**, 13505–13525 (2019).

[38] W. Cencek, K. Szalewicz, C. Leforestier, R. van Harrevelt, and A. van der Avoird, "An accurate analytic representation of the water pair potential," Phys. Chem. Chem. Phys. **10**, 4716–4731 (2008).
